# Emodin Inhibits NLRP3 Inflammasome Activation and Protects Against Sepsis via Promoting FUNDC1-Mediated Mitophagy

**DOI:** 10.7150/ijbs.110904

**Published:** 2025-05-27

**Authors:** Wei Fu, Shu-chang Liu, Tong-xiang Xu, Ying Liu, Teng Zhang, Dong-jie Liu, Xiao Wang, Jian-yao Wang, Yu-xin He, Tao Ma

**Affiliations:** 1 Department of Integrative Chinese and Western Medicine, Tianjin Medical University General Hospital, Tianjin, 300152, China.; 2 Unit of Infection and Immunity, Tianjin Medical University General Hospital Institute of General Surgery, Tianjin, 300152, China.; 3 Department of General Surgery, Tianjin Medical University General Hospital, Tianjin, 300152, China.; 4 Department of Integrative Chinese and Western Medicine, Tianjin First Center Hospital, Tianjin, 300192, China.

**Keywords:** Emodin, NLRP3 inflammasome, Mitochondrial homeostasis, Mitophagy, FUNDC1, Sepsis.

## Abstract

Dysregulated activation of the NLR family pyrin domain-containing 3 (NLRP3) inflammasome contributes to the pathogenesis of numerous inflammatory and infectious diseases; however, effective targeted therapies remain elusive. In this study, we identify emodin—a bioactive anthraquinone derived from *Rheum palmatum* (radix Rhei) and *Polygonum cuspidatum* (Polygonaceae)—as a potent and selective inhibitor of NLRP3 inflammasome activation. Notably, emodin disrupts the assembly of the NLRP3 complex without impairing inflammasome priming. Transcriptomic profiling via RNA sequencing reveals that emodin reprograms mitochondrial quality control pathways, markedly enhancing mitophagy flux. Mechanistically, emodin suppresses casein kinase II (CK2)-mediated phosphorylation of FUNDC1, a pivotal mitophagy receptor, thereby promoting mitochondrial clearance and preventing mitochondrial reactive oxygen species-induced NLRP3 inflammasome assembly. Both genetic silencing of FUNDC1 and pharmacological inhibition of mitophagy with 3-methyladenine abrogated abrogate the inhibitory effects of emodin, establishing a direct mechanistic link between FUNDC1-dependent mitophagy and NLRP3 regulation. *In vivo*, emodin confers significant protection in sepsis models, with these protective effects being lost in NLRP3-deficient mice or upon macrophage-specific deletion of FUNDC1. Collectively, our findings uncover a novel CK2-FUNDC1-mitophagy axis through which emodin inhibits NLRP3 inflammasome activation, highlighting its promise as a clinically translatable candidate for the treatment of NLRP3-driven inflammatory diseases.

## Introduction

The NLR family pyrin domain (PYD)-containing 3 (NLRP3) inflammasome functions as a pivotal cytoplasmic surveillance complex, consisting of three essential components: the sensor molecule NLRP3, the adaptor protein apoptosis-associated speck-like protein containing a CARD (ASC), and the effector enzyme caspase-1 1. Acting as a sentinel of intracellular stress, the NLRP3 inflammasome detects a wide array of danger-associated molecular patterns (DAMPs) and pathogen-associated molecular patterns (PAMPs) generated during microbial invasion or sterile tissue injury 2. Its activation follows a well-defined two-step signaling model. The initial priming phase, initiated by PAMPs (e.g., lipopolysaccharide) or pro-inflammatory cytokines (e.g., tumor necrosis factor alpha [TNF-α]), induces nuclear factor kappa B (NF-κB)-mediated transcriptional upregulation of pro-interleukin-1 beta [IL-1β], pro-IL-18, and NLRP3 itself, thereby licensing the inflammasome for activation 3-5. A subsequent activation signal, such as extracellular adenosine triphosphate (ATP), nigericin, or monosodium urate crystals, then promotes NLRP3 oligomerization and ASC speck formation via PYD interactions. This process enables the recruitment of pro-caspase-1 to ASC's CARD domain, facilitating its auto-proteolytic cleavage into active caspase-1 6, 7. The ensuing cascade culminates in the maturation and secretion of the pro-inflammatory cytokines IL-1β and IL-18, which orchestrate host defense mechanisms essential for pathogen clearance and tissue repair 8-11. However, dysregulated, or persistent activation of the NLRP3 inflammasome drives pathological inflammation, contributing to tissue injury and organ dysfunction in a range of diseases including sepsis, gout, acute peritonitis, and neurodegenerative disorders 12, 13. Therefore, precise regulation of NLRP3 inflammasome activity is indispensable for maintaining immune homeostasis, striking a critical balance between effective host defense and the prevention of excessive tissue damage 14.

Beyond their canonical role in cellular bioenergetics, mitochondria have emerged as dynamic signaling organelles that integrate metabolic stress cues to modulate immune responses 15. Accumulating evidence highlights mitochondrial dysfunction as a central driver of NLRP3 inflammasome activation. Pathological stimuli— including microbial infection, metabolic perturbations, and oxidative stress—induce mitochondrial damage and trigger the release of DAMPs. Notably, mitochondrial reactive oxygen species (mtROS) have been identified as key mediators that facilitate the assembly of the NLRP3 inflammasome complex 14, 16, 17. This mechanistic link is reinforced by pharmacological studies demonstrating that scavenging mtROS with antioxidants markedly attenuates NLRP3 activation 18-20, thus establishing mitochondrial oxidative stress as a proximal trigger of inflammasome signaling. Complementing this pathway, mitophagy— a selective form of autophagy targeting damaged mitochondria— acts as an intrinsic brake on NLRP3 activation. By eliminating dysfunctional mitochondria and limiting mtROS accumulation, mitophagy curbs aberrant inflammasome activity. Disruption of mitophagy, either genetically or pharmacologically, leads to mtROS accumulation, exacerbating NLRP3 activation and promoting inflammatory disease pathogenesis 21-24. Conversely, pharmacological enhancement of mitophagy suppresses mtROS production, restrains inflammasome activation, and alleviates inflammation in preclinical models. Collectively, these findings position mitophagy as a promising therapeutic axis for fine-tuning NLRP3-driven inflammation across diverse pathological contexts, including sepsis and neurodegenerative diseases 20, 25, 26.

Mitophagy, the autophagic clearance of damaged mitochondria, plays an essential role in maintaining cellular redox balance and preventing excessive ROS buildup. This process is orchestrated by intricate molecular networks that govern mitochondrial recognition and autophagosome formation 27-30. Central to this machinery is FUNDC1, an outer mitochondrial membrane receptor containing an N-terminal LC3-interacting region motif, which directly recruits autophagosome LC3 to initiate mitophagosome biogenesis. Emerging evidence suggests a functional interplay between FUNDC1-mediated mitophagy and NLRP3 inflammasome regulation 31-33. Specifically, FUNDC1-driven mitochondrial quality control has been shown to reduce IL-1β secretion in macrophages, inhibit hepatocellular carcinoma progression, and confer cardio-protection following ischemic injury— effects attributed to suppression of NLRP3 activation 33-35. Notably, FUNDC1 activity is tightly regulated via post-translational modifications. Casein kinase II (CK2), a constitutively active serine/threonine kinase, phosphorylates FUNDC1 at serine 13 (Ser13), sterically hindering its interaction with LC3 and thus inhibiting mitophagy 36. Conversely, dephosphorylation of Ser13 enhances FUNDC1-LC3 binding, promoting mitochondrial clearance and reducing ROS levels. This phosphorylation-dependent molecular switch provides a rapid and adaptable means to regulate mitophagy flux in response to cellular stress. Consequently, pharmacological modulation of FUNDC1 phosphorylation emerges as a promising strategy to control NLRP3 inflammasome activity and mitigate its pathological consequences 31.

**Figure 1:**
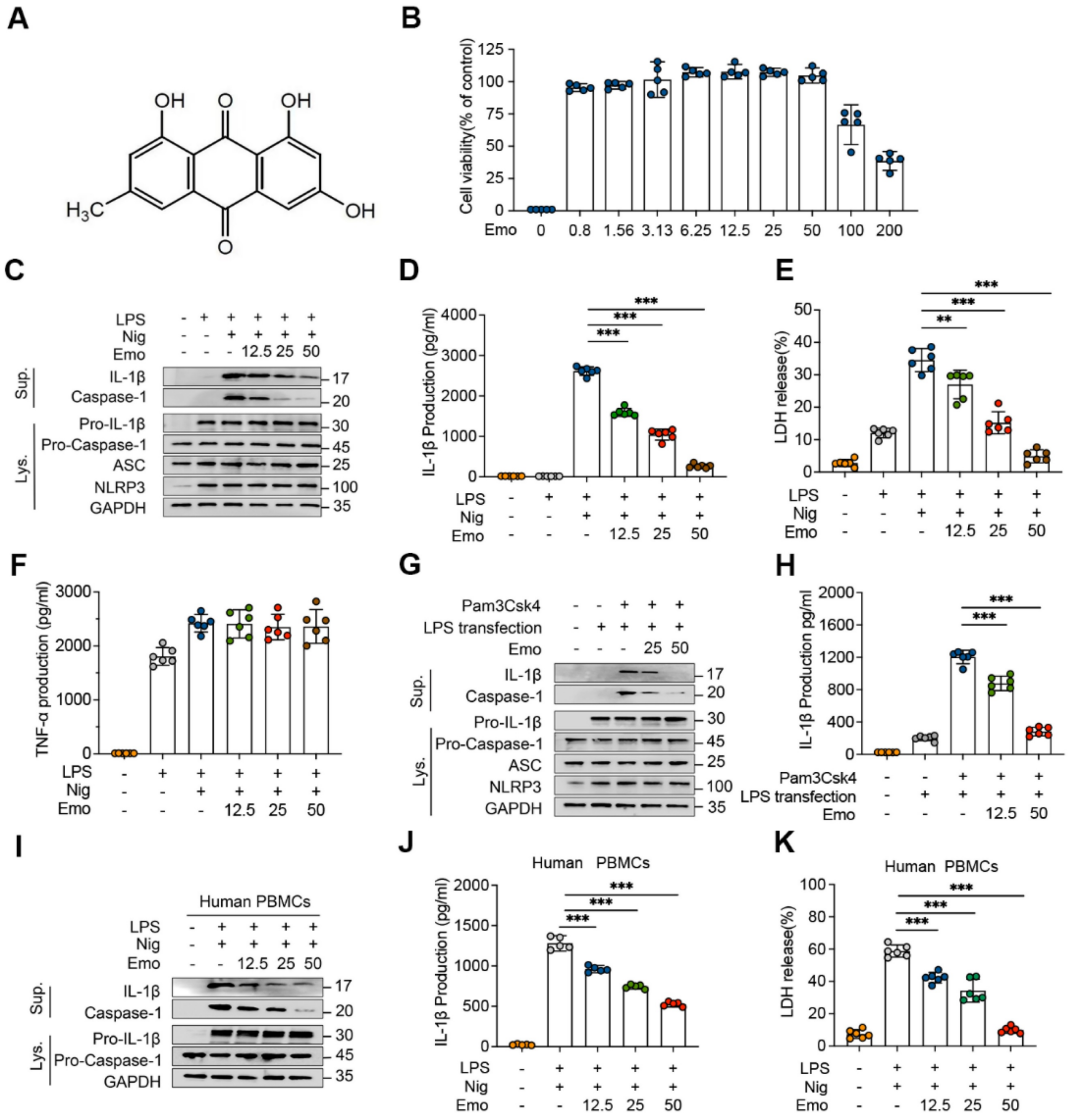
Emodin inhibits NLRP3 inflammasome activation in both BMDMs and human PBMCs. (A) Chemical structure of emodin (Emo). (B) BMDMs maintained viability after treatment with various doses of emodin (n = 5 per group). (C-F) Western blot analysis reveals alterations in NLRP3, pro-caspase-1, ASC, and pro-IL-1β expression in lysates (Lys) and IL-1β (p17), caspase-1 (p20) in supernatants (Sup) of BMDMs treated with emodin. The levels of IL-1β, LDH, and TNF-α in BMDM were quantified (n = 6 per group). (G-H) Western blot analysis reveals alterations in NLRP3, pro-caspase-1, ASC, and pro-IL-1β expression in Lys and IL-1β (p17), caspase-1 (p20) in Sup of BMDMs treated with emodin (G). Pam3CSK4 and transfected with LPS after emodin and IL-1β levels in Sup (n = 6 per group) (H). (I-K) Western blot analysis reveals alterations in pro-caspase-1 and pro-IL-1β expression in Lys and IL-1β (p17), caspase-1 (p20) in Sup of PBMCs treated with emodin(I). Quantification revealed reduced IL-1β (J) and LDH (K) levels in Sup (n = 6 per group). Data are presented as mean ± SEM from three independent experiments with statistical significance at ***P<0.001.

Emodin (1,3,8-trihydroxy-6-methylanthraquinone; Fig. 1A), a major active compound extracted from *Rheum palmatum* (radix Rhei) and *Polygonum cuspidatum* (Polygonaceae), embodies the pharmacological legacy of traditional Chinese medicine (TCM) 37. These botanicals have been extensively utilized in TCM formulations such as the Da-Cheng-Qi decoction, historically prescribed for the treatment of gastrointestinal disorders, infectious diseases, and inflammatory conditions 38, 39. Modern pharmacological research has elucidated the multifaceted bioactivity of emodin, including potent anti-inflammatory, antimicrobial, antioxidant, and anticancer effects. Mechanistically, emodin exerts its immunomodulatory functions by inhibiting the NF-κB signaling pathway, thereby suppressing the production of pro-inflammatory cytokines such as TNF-α and IL-6 40, 41. Beyond its established role in NF-κB modulation, recent studies have implicated emodin in the inhibition of NLRP3 inflammasome activation, leading to decreased IL-1β secretion and attenuation of the inflammatory cascade 42. However, the precise molecular targets and cytoprotective mechanisms underlying emodin's inflammasome-suppressive effects remain incompletely defined. Intriguingly, emodin has been identified as a selective inhibitor of CK2, binding competitively to the ATP-binding pocket of the kinase 43. Given the emerging link between CK2 activity and NLRP3 inflammasome regulation, a critical knowledge gap persists regarding whether emodin exerts its anti-inflammatory effects through modulation of FUNDC1- mediated mitophagy, a downstream effector pathway of CK2 signaling.

In this study, we demonstrate that emodin potently suppresses NLRP3 inflammasome activation and delineate a novel mechanistic pathway whereby emodin enhances mitochondrial quality control via CK2-dependent dephosphorylation of FUNDC1, thereby promoting mitophagy. *In vivo*, emodin confers significant protection in sepsis models, with these protective effects being lost in NLRP3-deficient mice or upon macrophage-specific deletion of FUNDC1. Collectively, our findings provide compelling evidence that emodin effectively attenuates NLRP3-driven inflammation, establishing it as a promising candidate for the prevention of inflammatory and infectious diseases associated with aberrant NLRP3 inflammasome activation.

## Materials and Methods

### Animals

C57BL/6 wild-type (WT) mice and NLRP3 knockout (NLRP3^-/-^) mice, weighing between 20 and 25 grams and aged 6 to 8 weeks, were procured from Beijing SPF Biotechnology Co., Ltd. (Beijing, China) and Shanghai Model Organisms Center, Inc. (Shanghai, China), respectively. The mice were maintained in a SPF facility before the commencement of experimental procedures. All animal experimentation was conducted in strict adherence to the guidelines and protocols sanctioned by the Animal Care and Use Committee of the Scientific Research Board at Tianjin Medical University (IRB2022-DWFL-472).

### Reagents and antibodies

The reagents utilized in this study were sourced from various suppliers as follows: Sigma-Aldrich (Munich, Germany) provided lipopolysaccharide (LPS, Escherichia coli strain 0111:B4, L2630), emodin (E7881), adenosine triphosphate (ATP, A1852), and dimethyl sulfoxide (DMSO, D2650). InvivoGen (Toulouse, France) supplied nigericin (tlrl-nig), monosodium urate (MSU, tlrl-msu), Pam3CSK4 (tlrl-pms), poly(dA:dT) (tlrl-patn), and flagellin (tlrl-stfla). MitoTracker (M7514, M22426) and MitoSOX (M36008) were obtained from Invitrogen (Carlsbad CA, USA). Adipogen (San Diego, CA, USA) furnished anti-mouse caspase-1 (1:1000 dilution, AG-20B-0042) and anti-NLRP3 (1:1000, AG-20B-0014). Anti-mouse IL-1β (1:1000, AF-401-NA) was acquired from R&D Systems (Minneapolis, MN, USA), while Santa Cruz Biotechnology Inc. (Dallas, TX, USA) provided anti-ASC (1:1000, sc-22514-R). Anti-human caspase-1 (1:1000, 2225S), anti-human IL-1β (1:1000, 12703), anti-human CK2α (1:1000,2656), and anti-LC3A/B (1:1000, 12741T) were sourced from Cell Signaling Technology (Boston, MA, USA). ABclonal (Wuhan, China) supplied antibodies against FUNDC1 (1:1000, a22001), LC3B (1:1000, a196665), and Flag (1:1000, AE005), as well as secondary antibodies: ABflo 488-conjugated goat anti-mouse IgG (1:1000, AS037), ABflo 555-conjugated goat anti-mouse IgG (1:1000, AS057), and ABflo Cy3-conjugated goat anti-rabbit IgG (1:1000, AS007). Anti-GAPDH (1:1000, C1312) was purchased from Pulilai Gene Technology (Beijing, China), and a customized anti-p-FUNDC1 antibody (1:1000) was produced by Abiocenrer (Beijing, China).

### Cell culture

Bone marrow-derived macrophages (BMDMs) were extracted from the femur bone marrow of 6-week-old male C57BL/6 mice and subsequently cultured in DMEM containing 50 ng/mL of M-CSF for 1 week 44. Peripheral blood mononuclear cells (PBMCs) were isolated from freshly drawn peripheral venous blood utilizing Human Lymphocyte Separation Medium, adhering to the manufacturer's protocol. Prior to any stimulation, these PBMCs were allowed to adapt in RPMI 1640 medium overnight. Human THP-1 cells, sourced from Suzhou Cyagen Biosciences Inc., were maintained in RPMI 1640, and differentiated using 100 nM PMA overnight. All cell cultures were maintained in a 37°C, 5% CO_2_ humidified incubator (HEARcell 150i by Thermo Fisher Scientific).

### Inflammasome activation

BMDMs, THP-1 cells, and PBMCs were plated in 6-well plates at concentrations of 1 × 10^5^ cells/mL, 5 × 10^5^ cells per well, and 2.5 × 10^5^ cells per well, respectively, and left to attach overnight. On the subsequent day, the medium was changed, and the cells were stimulated with 50 ng/mL of LPS for a duration of 4 h. Afterward, the medium was replaced with Opti-MEM containing emodin for 1 h. To activate the canonical NLRP3 inflammasome, the cells were exposed to nigericin at 10 μM for 30 min, ATP at 5 mM for 45 min, or MSU at 200 μg/mL for 6 h. For NLRC4 inflammasome activation, the cells were treated with Lfn-Flic at 200 ng/mL for 6 h. To induce AIM2 inflammasome activation, poly(dA:dT) at 2 μg/mL was transfected into the cells using Lipofectamine 2000 for 6 h. For noncanonical NLRP3 inflammasome activation, BMDMs were first primed with Pam3CSK4 at 1 μg/mL for 4 h, followed by replacement of the medium with Opti-MEM containing emodin for 1 hour, and subsequently transfected with 1 μg/mL of LPS using Lipofectamine 2000 for 6 h.

### RNA sequencing and bioinformatics analyses

Cellular RNA was extracted from BMDMs using the TRIzol reagent (Invitrogen, USA). Samples that passed the basic quality metrics were used for RNA-Seq analysis. The sequencing data quality was evaluated using FastQC (version 0.11.5), followed by filtering out poor-quality sequences using NGSQC (version 2.3.3). StringTie (version 1.3.3b) was utilized for gene expression analysis, while DESeq2 (version 1.40.2) was employed to identify differentially expressed genes (DEGs) across the samples. The criteria for identifying DEGs included a ≥ 2-fold change in transcript abundance (|log2FC| ≥ 1) and an adjusted *P*-value < 0.01. The volcano plot and heatmap were generated using R4.3.1 software. Gene ontology (GO) functional enrichment analysis and gene set enrichment analysis (GSEA) were performed using the R package 'cluster Profiler'. The M2 and M5 gene sets were downloaded from the MSigDB database. Subsequently, the gene sets were identified as significantly enriched after normalization, with a *P* < 0.05 and a false discovery rate (FDR) < 0.25.

### *In vivo* LPS challenge

C57BL/6 and NLRP3^-/-^ mice were administered 20 mg/kg emodin or vehicle control via intraperitoneal injection 1 h prior to 10 mg/kg LPS or PBS. The levels of IL-1β and TNF-α in peritoneal lavage fluid (PLF) and serum were detected using ELISA 8 h after LPS treatment. Lung and liver tissue sections underwent hematoxylin and eosin staining 24 h following LPS injection. Additional lung sections were harvested for quantification of NLRP3 and mitochondrial autophagy-related protein expression and western blot.

### Adeno-associated virus gene construction and infection of C57BL/6 mice

An adeno-associated virus (AAV) vector carrying a macrophage-specific FUNDC1 deletion plasmid was generated by GeneChem Co., Ltd. (Shanghai, China). The AAV9 system incorporates a macrophage-specific synthetic promoter, F4/80p, driving the expression of short hairpin RNA (shRNA) specifically targeting FUNDC1 based on miR155 sequences, along with an enhanced green fluorescent protein (EGFP) reporter. The nucleotide shRNA of FUNDC1 were cloned using the following sequence: 5'-GCAGCACCTGAAATCAACAAT-3', 5'-CCACTGGTGGAATCGAG TATT- 3', and 5'-AGGTG GTGGTTTCCTTCTTCT-3'. The recombinant vector was verified by DNA sequencing to confirm the accuracy of the cloned sequences. Four-week-old male mice were injected via the tail vein with AAV-shFUNDC1 or AAV-Ctrl shRNA virus at a dosage of 1 × 10^12^ vector genomes per mouse diluted in 50 μl of PBS. On day 21 post-injection, GFP expression in infected macrophages was analyzed using flow cytometry, and FUNDC1 expression was evaluated with western blot.

### Statistical analysis

Data analysis was carried out using GraphPad Prism version 8.0.2. The experimental outcomes are presented as mean ± standard error of the mean. An unpaired two-tailed t-test was applied for comparisons between two groups. For experiments with multiple groups and a single independent variable, one-way analysis of variance was used, followed by the Bonferroni post-hoc test to determine statistical significance. The log-rank (Mantel-Cox) test was applied for survival analysis. Statistical significance was defined as a *P*-value < 0.05. * *P* < 0.05, ** *P* < 0.01, *** *P* < 0.001.

Additional details for all methods are provided in the Supplementary Methods.

## Results

### Emodin suppresses both canonical and noncanonical activation of the NLRP3 inflammasome

The cytotoxicity of emodin in BMDMs was assessed using the CCK-8 assay. Concentrations up to 50 μM did not significantly affect cell viability compared to untreated controls (Fig. 1B), supporting their selection for subsequent experiments. To investigate the inhibitory effects of emodin on NLRP3 activation, BMDMs were primed with LPS, pretreated with emodin (12.5-50 μM), and subsequently stimulated with nigericin. Immunoblot analysis revealed a dose-dependent decrease in secreted mature IL-1β and cleaved caspase-1 (Fig. 1C), indicating that emodin suppresses NLRP3-mediated caspase-1 activation. Notably, emodin did not alter intracellular levels of NLRP3, ASC, pro-caspase-1, or pro-IL-1β (Fig. 1C), suggesting its action is confined to the activation stage. Consistently, emodin significantly reduced the release of IL-1β and lactate dehydrogenase (LDH) into the supernatant (Fig. 1D and E). To confirm inflammasome-specific effects, we measured TNF-α secretion, a cytokine dependent on priming but independent of inflammasome activation. Emodin had no effect on TNF-α levels (Fig. 1F), validating its selective inhibition of NLRP3-mediated IL-1β processing. To evaluate the broad-spectrum inhibitory potential of emodin against NLRP3 activation, BMDMs were stimulated with alternative NLRP3 activators, including ATP and MSU. Emodin suppressed IL-1β release and caspase-1 cleavage in a dose-dependent manner (Fig. S1A and B, D and E), without affecting TNF-α production (Fig. S1C, F), demonstrating its efficacy across diverse NLRP3 stimuli. We further explored emodin's impact on noncanonical NLRP3 activation, wherein intracellular LPS activates caspase-11, leading to IL-1β maturation 45. Emodin pretreatment dose-dependently inhibited caspase-1 cleavage and IL-1β secretion following LPS transfection (Fig. 1G and H), indicating that emodin suppresses both canonical and noncanonical pathways of NLRP3 activation. To assess specificity, we examined the effects of emodin on the AIM2 and NLRC4 inflammasomes. BMDMs were primed with LPS and subsequently stimulated with poly(dA:dT) (an AIM2 activator) or transfected with Lfn-Flic (an NLRC4 activator). Emodin did not affect IL-1β or LDH release (Fig. S2B and C, E and F), nor did it inhibit caspase-1 cleavage (Fig. S2A, D), confirming its specificity for the NLRP3 inflammasome.

Finally, to validate translational relevance, we tested emodin in human PBMCs from healthy donors. Consistent with murine BMDMs, emodin dose-dependently suppressed caspase-1 cleavage, IL-1β secretion, and LDH release (Fig. 1I-K). Collectively, these results establish emodin as a selective NLRP3 inflammasome inhibitor with potential translational application in NLRP3-driven human diseases.

### Emodin does not inhibit NLRP3 priming but disrupts ASC oligomerization during NLRP3 activation

**Figure 2:**
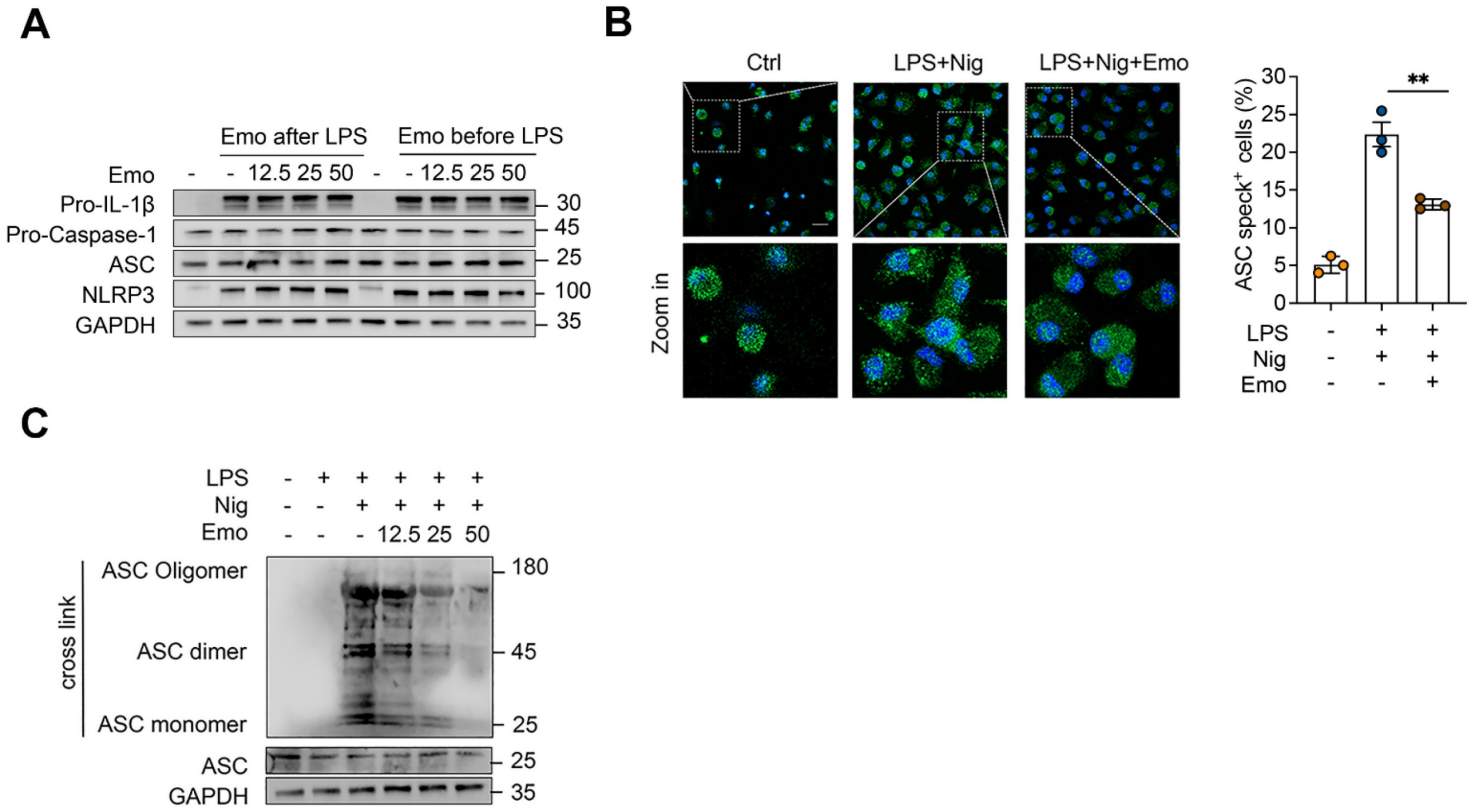
Emodin does not suppress NLRP3 priming but impedes NLRP3-mediated ASC oligomerization. (A) Western blot analysis was conducted on the specified proteins in Lys from BMDMs that were either exposed to LPS for 4 h prior to treatment with varying concentrations of emodin for 1 h (emodin post-LPS) or pretreated with different doses of emodin for 1 h followed by LPS stimulation for 4 h (emodin pre-LPS). (B) Immunofluorescence images depict the subcellular localization of ASC (green) in LPS-primed BMDMs stimulated with nigericin, with or without emodin. Nuclei are counterstained with DAPI (blue); scale bar: 20 μm. The proportion of cells exhibiting ASC specks (green) in BMDMs was quantified. (C) Western blot analysis was carried out to assess cross-linked ASC in BMDMs subjected to the same treatment as in (B). Data are expressed as the mean ± SEM from three independent experiments, with biological duplicates for (B), or are representative of three independent experiments for (A, C). **P<0.01 vs the control.

Activation of the NLRP3 inflammasome involves a two-step process: a priming phase, in which pro-IL-1β and NLRP3 is upregulated, followed by an activation phase that triggers pro-caspase-1 cleavage and IL-1β maturation 8. To determine whether emodin influences the priming step, BMDMs were treated with 12.5, 25, or 50 μM emodin either before or after LPS priming. Western blot analysis revealed no notable changes in the protein levels of NLRP3, ASC, pro-caspase-1, or pro-IL-1β under any condition, confirming that emodin does not impair LPS-induced priming at concentrations sufficient to inhibit inflammasome activation (Fig. 2A). During the activation phase, NLRP3 promotes ASC speck formation through PYD-PYD interactions, a critical step in inflammasome assembly 1. Immunofluorescence microscopy revealed robust perinuclear ASC speck formation in activated cells, which was markedly attenuated by emodin pretreatment (Fig. 2B). Consistently, western blot analysis further confirmed that emodin significantly suppresses nigericin-induced ASC oligomerization (Fig. 2C). Collectively, these findings indicate that emodin selectively disrupts ASC polymerization and inflammasome assembly, while leaving the priming phase intact.

### Emodin preserves mitochondrial homeostasis during NLRP3 inflammasome activation

**Figure 3:**
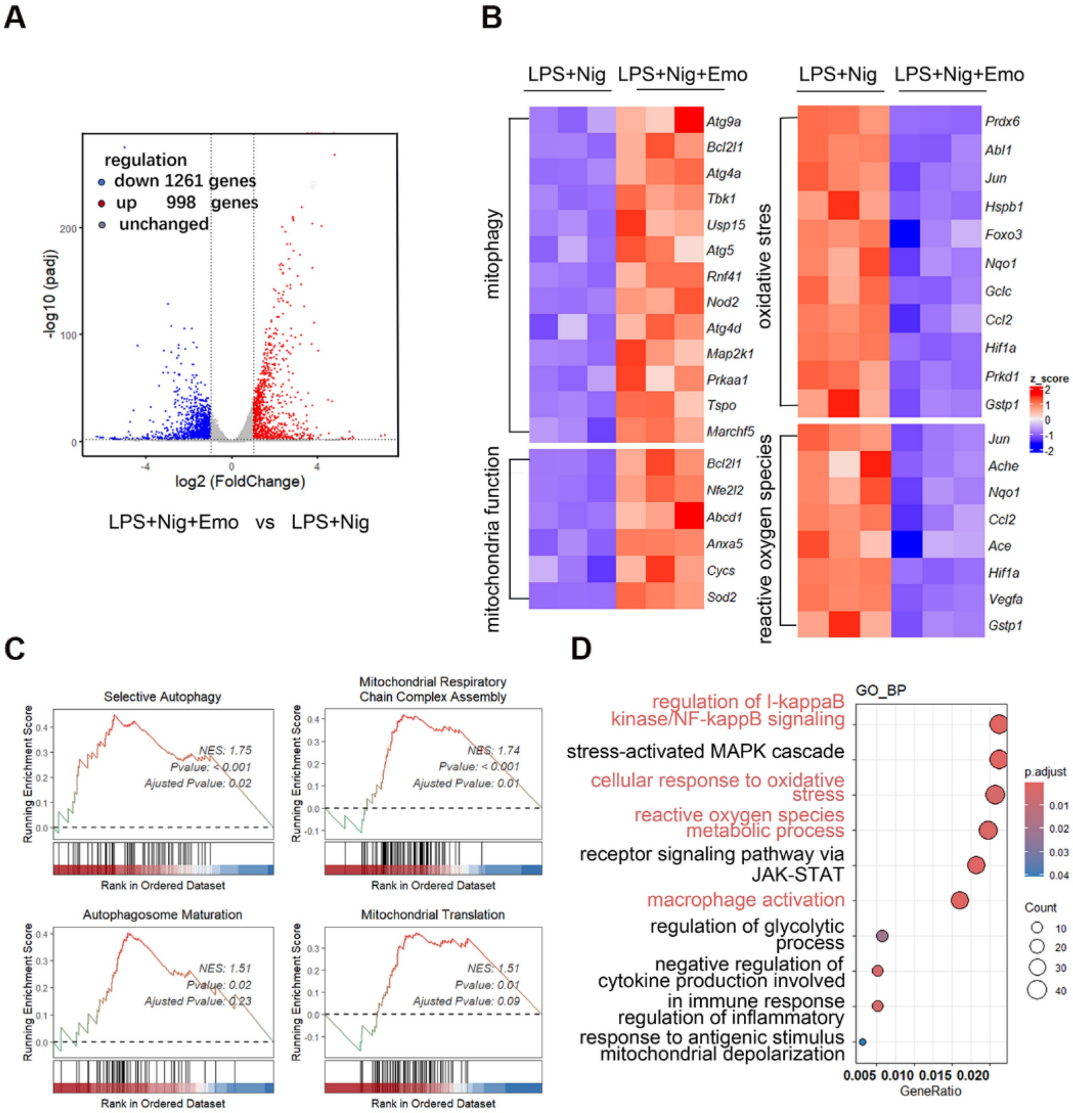
Emodin modulates mitochondrial homeostasis and mitophagy upon NLRP3 inflammasome activation. (A) Volcano plots illustrate the differential gene expression between the LPS + nigericin and LPS + nigericin + emodin treatment groups. Genes up-regulated and down-regulated are highlighted in red and blue, respectively. (B) A heatmap presents the relative mRNA expression of genes associated with mitophagy, mitochondrial function, oxidative stress, and reactive oxygen species in the two treatment groups: LPS + nigericin and LPS + nigericin + emodin. (C) Gene Set Enrichment Analysis (GSEA) was conducted to identify significantly up-regulated genes in the LPS + nigericin + emodin group versus the LPS + nigericin group. (D) GO Pathway Enrichment Analysis (focusing on Biological Process, BP) was performed based on the differentially expressed genes (DEGs).

To elucidate the mechanism by which emodin modulates NLRP3 inflammasome activation, we performed transcriptomic profiling of BMDMs primed with LPS and stimulated with nigericin, with or without emodin pretreatment. Volcano plot analysis identified 2,259 differentially expressed genes (DEGs, *P* < 0.01) in emodin-treated cells relative to NLRP3-activated controls, including 998 upregulated and 1,261 downregulated genes (Fig. 3A). Notably, emodin-upregulated genes were enriched in pathways related to mitochondrial function maintenance (*Sod2, Cycs, Anxa5*), oxidative stress response (*Hifa, Foxo3, Abl1*), mitophagy regulation (*Atg9a, Atg4a, Atg5*), and ROS metabolism (*Jun, Ache, Ccl2*) (Fig. 3B). GSEA further demonstrated significant enrichment of emodin-upregulated genes in pathways associated with mitochondrial respiratory chain complex assembly, autophagosome maturation, selective autophagy, and mitochondrial translation, with normalized enrichment scores (NES) of 1.74, 1.51, 1.75, and 1.51, respectively (Fig. 3C). GO enrichment analysis underscored the involvement of DEGs in immune regulation and oxidative stress management (Fig. 3D). Collectively, these data suggest that emodin reinforces mitochondrial homeostasis during NLRP3 inflammasome activation.

Beyond their role in energy metabolism, mitochondria are increasingly recognized as central regulators of NLRP3 activation. Mitochondrial dysfunction and subsequent mtROS accumulation have been identified as key triggers of NLRP3 inflammasome activation 46. Transcriptome analysis implied that emodin promotes mitochondrial integrity. To experimentally validate this, we quantified mtROS production using MitoSOX Red™ staining. As expected, LPS/nigericin stimulation elevated mtROS levels, whereas emodin pretreatment markedly reduced mtROS accumulation (Fig. S3A and B). Furthermore, dual MitoTracker staining (Deep Red for active mitochondria, Green for total mitochondrial mass) revealed that NLRP3 activation increased the proportion of damaged mitochondria, which was significantly diminished by emodin treatment (Fig. S3C and D). These findings substantiate the transcriptomic predictions and demonstrate that emodin preserves mitochondrial homeostasis during NLRP3 inflammasome activation.

### Emodin promotes mitophagy, thereby suppressing NLRP3 inflammasome activation

**Figure 4:**
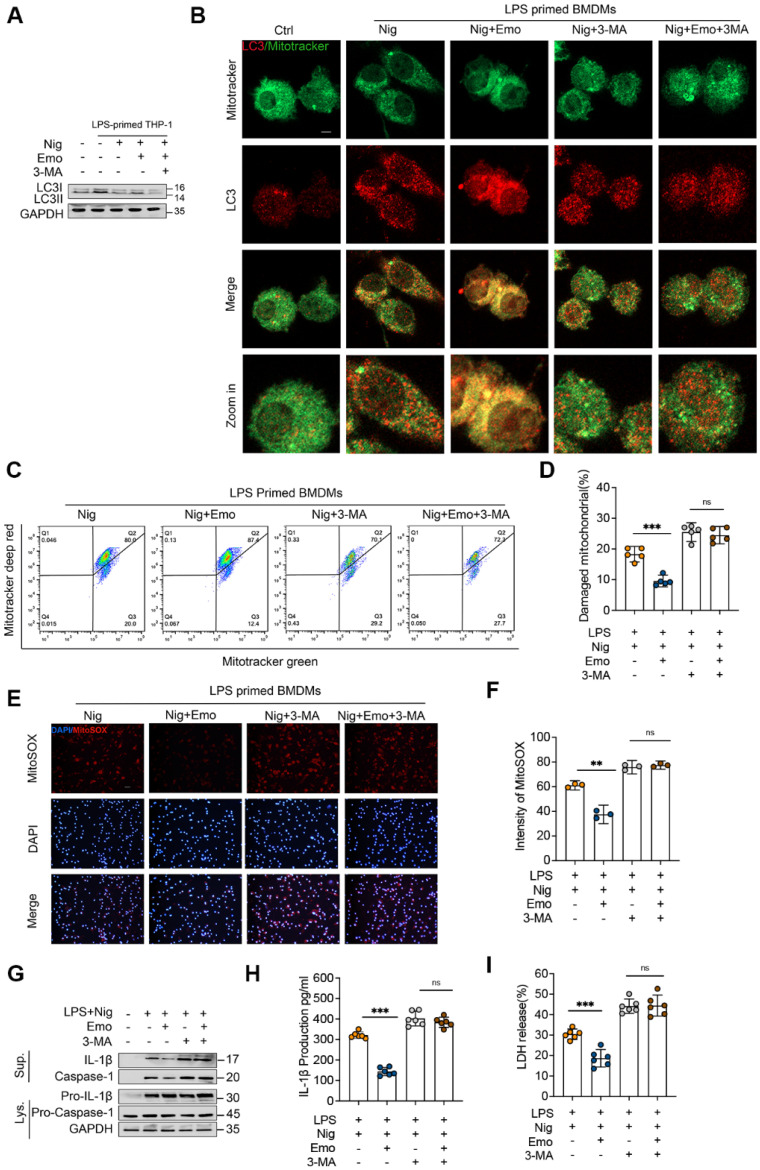
Emodin enhances mitophagy to preserve mitochondrial homeostasis, subsequently inhibiting NLRP3 inflammasome activation. BMDMs primed with LPS were pretreated with 3-MA (5mM for 1 h) prior to emodin treatment (25μM for 1 h), followed by nigericin stimulation (10 μM for 30 min). (A) Western blot analysis was conducted on the specified proteins in Lys. (B) Confocal microscopy was employed to observe the colocalization of LC3 (red) with mitochondria (green). Scale bar: 10μm. (C-D) BMDMs were labeled with Mitotracker Green (for total mitochondria) and Mitotracker Deep Red (for respiring mitochondria), and subsequently analyzed using flow cytometry. (E-F) BMDMs were stained with MitoSOX and examined under a fluorescence microscope. Scale bar: 40μm. (G-I) Western blot analysis was performed to detect IL-1β (p17), caspase-1 (p20) in Sup, and pro-IL-1β (p30), caspase-1 (p45), NLRP3, and ASC in Lys (G). The levels of IL-1β (H) and LDH (I) in the Sup were quantified. Data are presented as mean ± SEM from three independent experiments with biological duplicates for (D, F, H, I), or are representative of three independent experiments for (A, G). **P<0.01 compared to control. ***P<0.001 compared to control. *ns*, not significant.

Mitophagy, an evolutionarily conserved protective mechanism, selectively eliminates damaged or dysfunctional mitochondria to reduce mtROS production and preserve mitochondrial homeostasis 47, 48. Our bioinformatics analysis indicated that emodin upregulates the expression of genes associated with selective autophagy and autophagosome maturation (Fig. 3A-D). Based on these findings, we investigated whether emodin modulates NLRP3 inflammasome activation via mitophagy enhancement. During autophagosome formation, cytosolic LC3-I undergoes lipidation to generate LC3-II, a hallmark of autophagy induction 49. In BMDMs stimulated with LPS and nigericin, emodin treatment significantly elevated LC3-II expression (Fig. 4A). Correspondingly, emodin markedly promoted the co-localization of mitochondria with LC3 in LPS/nigericin-stimulated BMDMs (Fig. 4B). To further substantiate the role of mitophagy in emodin-mediated suppression of NLRP3 activation, BMDMs were treated with 3-methyladenine (3-MA), a pharmacological inhibitor of mitophagy. As expected, 3-MA treatment significantly reduced LC3-II levels and abolished the emodin-induced enhancement of mitochondrial-LC3 co-localization, indicating impaired mitophagy (Fig. 4A and B).

Moreover, emodin treatment decreased the accumulation of damaged mitochondria in inflammasome-activated BMDMs, an effect that was effectively reversed by 3-MA co-treatment (Fig. 4C and D). Consistently, 3-MA administration led to a pronounced increase in MitoSOX-positive cells and elevated mtROS levels (Fig. 4E and F). Notably, the inhibitory effects of emodin on caspase-1 activation, IL-1β secretion, and LDH release were also markedly reversed by 3-MA treatment (Fig. 4G-I). Collectively, these findings suggest that emodin-induced mitophagy is a pivotal mechanism that eliminates damaged mitochondria, reduces mtROS production, restores mitochondrial homeostasis, and ultimately suppresses NLRP3 inflammasome activation.

### Emodin suppresses NLRP3 inflammasome activation by enhancing FUNDC1-dependent mitophagy

Mitophagy, a specialized form of autophagy targeting dysfunctional mitochondria, operates via two primary mechanisms: Parkin-dependent and receptor-mediated pathways. Among the latter, FUNDC1 serves as a crucial stress-responsive receptor that recruits LC3-II to mitochondria, facilitating mitophagy 50. Post-translational regulation of FUNDC1 by CK2-mediated phosphorylation at Ser13 critically modulates its activity— phosphorylation impairs LC3-II binding and inhibits mitophagy, whereas dephosphorylation promotes this process 36. Given the established CK2-inhibitory properties of emodin, we hypothesized that emodin may potentiate FUNDC1-dependent mitophagy by disrupting CK2-mediated phosphorylation.

**Figure 5:**
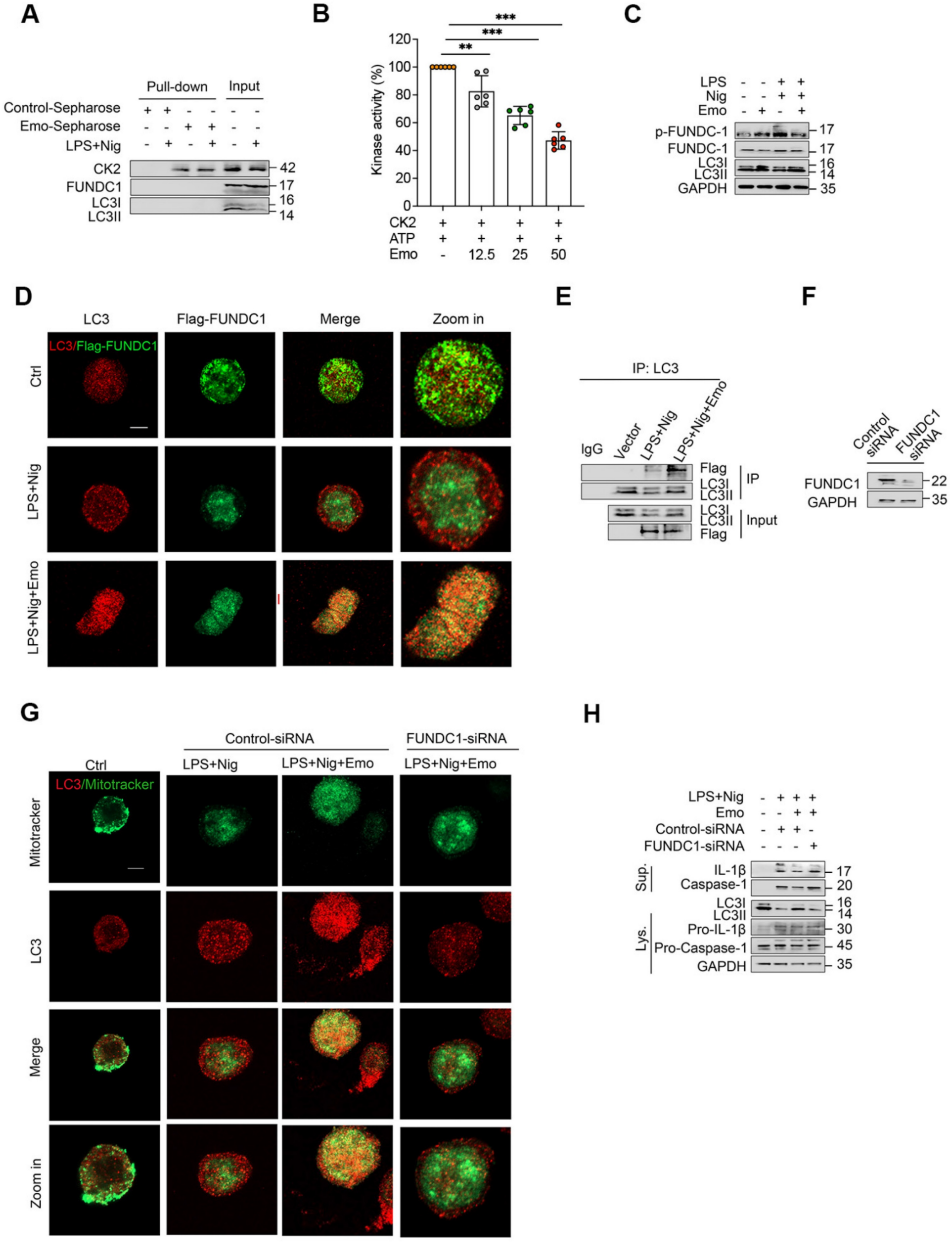
Emodin suppresses NLRP3 inflammasome activation via FUNDC1-mediated mitophagy. (A) Cell lysates of LPS-primed THP-1 with or without nigericin incubated with emodin-Sepharose. The pull-down samples and input were analyzed by western blot. (B) Effect of emodin on the ATPase activity of CK2. After incubation CK2 plus indicated different concentrations of free emodin (12.5 μM, 25 μM, and 50μM), ATP was measured by CellTiter-Glo Assay Kit and normalized to the control. (C) THP-1 cells, primed with LPS, were exposed to emodin or a vehicle control, followed by nigericin stimulation. Western blot analysis was conducted to assess levels of LC3II, FUNDC1, and p-FUNDC1. (D) THP-1 cells were transfected with Flag-tagged FUNDC1 and subjected to the same treatment as in (C). Confocal microscopy was used to examine the colocalization of LC3 (red) and Flag-FUNDC1 (green). Scale bars: 10 μm. (E) Cell Lys underwent immunoprecipitation with an anti-LC3 antibody, and subsequent western blot was performed to evaluate the interaction between LC3 and FUNDC1 in THP-1 cells treated as in (C). (D-F) THP-1 cells were transfected with FUNDC1 siRNA or a negative control siRNA for 24 h, followed by the treatment regimen outlined in the Figure. Western blot analysis was employed to measure FUNDC1 expression in Lys (D). Confocal microscopy was used to visualize the colocalization of LC3 (red) and Mitotracker (green). Scale bars: 10 μm (E). Western blot analysis was conducted to detect IL-1β (p17), caspase-1 (p20) in the Sup and pro-IL-1β (p30), caspase-1 (p45), LC3II in Lys (F). The data presented are representative of three independent experiments. **P<0.01 compared to control. ***P<0.001 compared to control. *ns*, not significant.

To test this, we first assessed CK2 kinase activity and observed that emodin inhibited CK2 in a dose-dependent manner (Fig. 5A). Subsequently, we performed affinity purification using emodin-conjugated CNBr-activated Sepharose beads to capture emodin-binding proteins from BMDM lysates. Immunoblotting confirmed specific enrichment of CK2 in the precipitates, while FUNDC1, LC3-I, and LC3-II were absent (Fig. 5B), indicating a direct interaction between emodin and CK2, excluding non-specific binding to core mitophagy effectors. Further mechanistic investigations revealed that NLRP3 inflammasome activation promoted FUNDC1 phosphorylation at Ser13 in THP-1 cells, an effect significantly attenuated by emodin treatment (Fig. 5C). Concordant with enhanced mitophagy, emodin stabilized FUNDC1-LC3 complexes (Fig. 5D and E), elevated LC3-II expression, reduced mtROS accumulation, and improved mitochondrial quality control. To determine the essential role of FUNDC1 in emodin-induced mitophagy, we performed siRNA-mediated knockdown of FUNDC1 (Fig. 5F). FUNDC1 silencing abrogated the effects of emodin, as evidenced by impaired mitochondrial-LC3 co-localization (Fig. 5G), accumulation of damaged mitochondria and mtROS (Fig. S4A-D), and reversal of emodin's inhibitory effects on caspase-1 cleavage and IL-1β maturation (Fig. 5H). Together, these findings delineate a CK2-FUNDC1-mitophagy axis underlying NLRP3 inflammasome regulation. By directly inhibiting CK2, emodin promotes FUNDC1-mediated mitophagy, facilitates mitochondrial clearance, and effectively suppresses NLRP3 inflammasome activation.

### Emodin protects against LPS-induced sepsis via suppressing NLRP3 inflammasome activation

**Figure 6:**
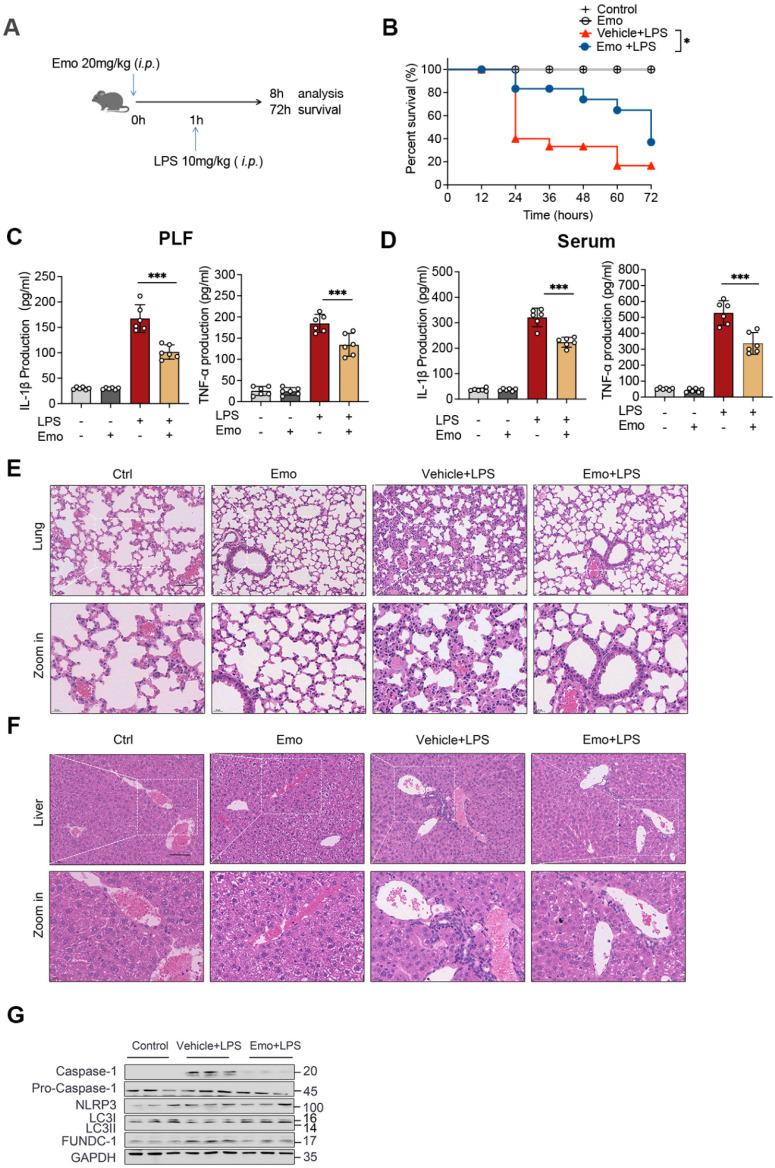
Emodin demonstrates protective efficacy against sepsis induced by LPS. (A) Schematic overview of the experimental design. (B) The survival rates of male C57BL/6 mice were monitored for 72 hours after intraperitoneal injection of LPS (10 mg/kg), with or without pretreatment with emodin (20 mg/kg). Statistical analysis was performed using the log-rank test. (C-D) Concentrations of IL-1β and TNF-α in serum (C) and PLF (D) were quantified using ELISA. (E-F) Representative H&E-stained histological images of lung (E) and liver (F) tissues are shown. Scale bar: 100 μm. (G) Western blot analysis was conducted to evaluate the protein expression levels of NLRP3, pro-caspase-1 (p45), caspase-1 (p20), and LC3II in lung tissue. Data are expressed as mean ± SEM or are representative of at least two independent experiments, with 5-7 animals per group. *P<0.05 compared to the control. ***P<0.001 compared to the control.

To validate the physiological relevance of our *in vitro* observations, we employed a well-established murine model of LPS-induced sepsis, which is driven by NLRP3 inflammasome-mediated inflammatory responses. Mice were pretreated with emodin via intraperitoneally 1 h prior to LPS challenge (Fig. 6A). Survival analysis revealed that emodin pretreatment significantly improved 72-hour survival rates compared to vehicle controls (Fig. 6B), aligning with prior findings. Quantification of inflammatory markers demonstrated substantial increases in IL-1β and TNF-α levels in both PLF and serum following LPS exposure, which were markedly attenuated by emodin treatment (Fig. 6C and D). Histopathological changes further confirmed these effects, showing severe lung and liver tissue damage in LPS-challenged mice, which was notably alleviated by emodin administration (Fig. 6E and F). Western blot analysis of lung tissues revealed that LPS stimulation induced robust upregulation of cleaved caspase-1, but significantly downregulated LC3II expression. These LPS-induced changes were reversed upon emodin treatment (Fig. 6G).

To determine whether emodin's protective effects were dependent on NLRP3, we utilized NLRP3 knockout (NLRP3^-/-^) mice. Genetic ablation of NLRP3 significantly suppressed IL-1β and TNF-α production in both PLF and serum, mitigated organ injury, and improved survival outcomes compared to WT controls after LPS challenge (Fig. S5A-F). Notably, emodin treatment provided minimal additional benefit in NLRP3^-/-^ mice across all assessed parameters (Fig. S5A-F), indicating that emodin's protective effects are predominantly mediated through NLRP3 inflammasome inhibition. Together, these *in vivo* findings establish that emodin confers protection against LPS-induced sepsis primarily by suppressing NLRP3 inflammasome activation, thereby dampening downstream inflammatory responses.

### FUNDC1-dependent mitophagy contributes to emodin-mediated inhibition of NLRP3 inflammasome activation in LPS-induced sepsis

**Figure 7:**
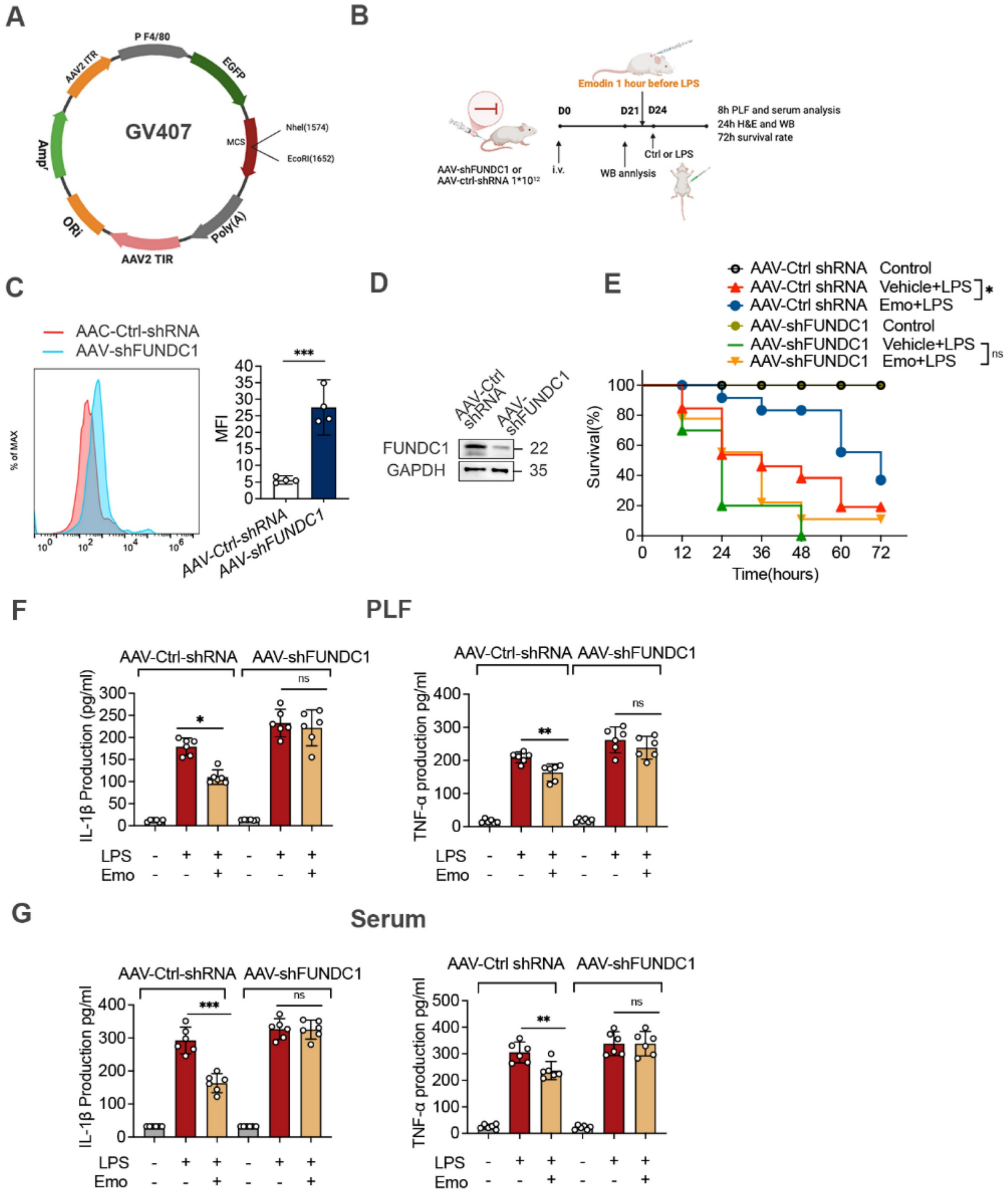

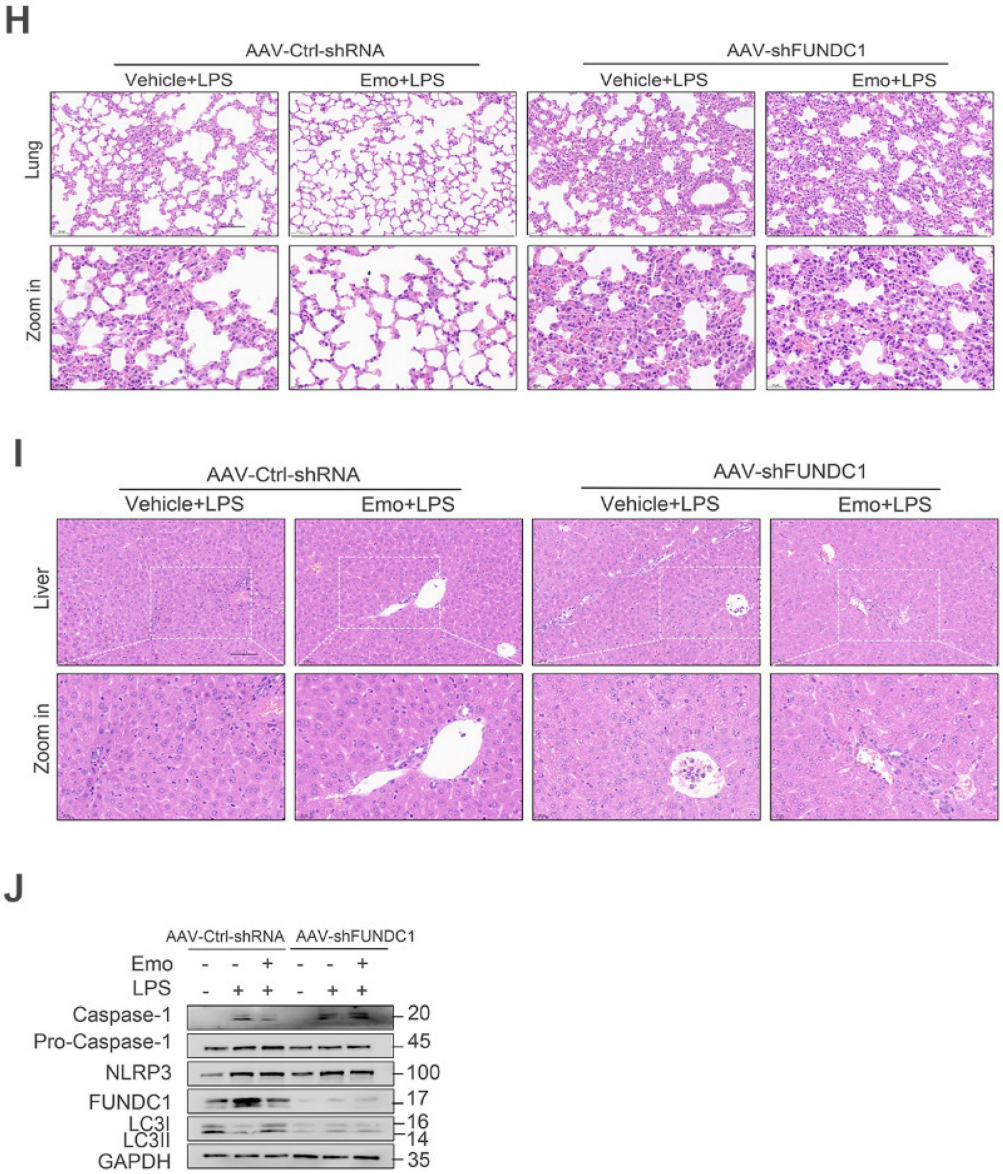
Emodin's effects on LPS-induced sepsis rely on FUNDC1-mediated mitophagy. (A) Schematic representation of the AAV-F4/80p-EGFP-MIR155(RNAi)-SV40-PolyA construct (AAV-shFUNDC1). (B) Experimental design overview: Mice were administered tail vein injections of either AAV-shFUNDC1 or AAV-Ctrl-shRNA. After 21 days, mice were pretreated with emodin (20 mg/kg), followed by an intraperitoneal injection of LPS (10 mg/kg) one hour later. (C) Flow cytometry was used to evaluate GFP expression in lung macrophages from mice infected with AAV-Ctrl-shRNA or AAV-shFUNDC1 on day 21. (D) Western blot analysis of FUNDC1 expression in lung tissue from mice infected with AAV-shFUNDC1 or AAV-Ctrl-shRNA. (E) Survival rates of male C57BL/6 mice were tracked over 72 hours. Statistical significance was assessed using the log-rank test. (F-G) IL-1β and TNF-α levels in serum (F) and PLF (G) were quantified by ELISA. (H-I) Representative H&E-stained images of lung (H) and liver (I) tissues. Scale bar: 100 μm. (J) Western blot analysis was conducted to measure the expression levels of NLRP3, pro-caspase-1 (p45), caspase-1 (p20), FUNDC1, and LC3II proteins in lung tissue. Data are expressed as mean ± SEM. Results are representative of at least two independent experiments, with 5-7 animals per group. *P<0.05 compared to control. **P<0.01 compared to control. ***P<0.001 compared to control. *ns*, not significant.

To elucidate the mechanisms underlying emodin's protective effects in sepsis, we investigated its role in regulating FUNDC1-mediated mitophagy in the lungs of septic mice. Western blot analysis revealed increased LC3II levels in the lungs of emodin-treated septic mice, indicative of enhanced mitophagy (Fig. 6G). To directly assess the contribution of FUNDC1, we employed an AAV system with a macrophage specific F4/80 promoter to deliver shRNA targeting FUNDC1 (Fig. 7A and B). Efficient delivery and specific knockdown of FUNDC1 in pulmonary macrophages were confirmed (Fig. 7C and D). Macrophage-specific FUNDC1 knockdown significantly impaired emodin's protective efficacy, as demonstrated by worsened organ injury and decreased survival in septic mice (Fig. 7E, H and I).

Furthermore, emodin-mediated reductions in IL-1β and TNF-α levels in both PLF and serum, as well as decreased cleaved caspase-1 expression in lung tissues, were reversed by FUNDC1 knockdown (Fig. 7F and G, J). Notably, FUNDC1 knockdown also abrogated emodin-induced upregulation of LC3II expression, indicating that emodin enhances mitophagy via a FUNDC1-dependent mechanism (Fig. 7J). In line with these findings, the inhibitory effects of emodin on NLRP3 inflammasome activation were nullified by FUNDC1 knockdown, as evidenced by increased caspase-1 cleavage and elevated IL-1β levels (Fig. 7F and G, J). Taken together, these results demonstrate that emodin protects against LPS-induced sepsis by promoting FUNDC1-dependent mitophagy, thereby suppressing NLRP3 inflammasome activation and mitigating the ensuing inflammatory response in mice.

## Discussion

The NLRP3 inflammasome has emerged as a pivotal molecular platform that not only functions as a frontline sentinel in host defense against pathogenic insults but also serves as a critical driver in the pathogenesis of a broad array of inflammatory diseases. This dual role encompasses a wide spectrum of clinical disorders, including systemic conditions such as sepsis and gout, acute inflammatory syndromes, and chronic neurodegenerative diseases 1, 51, 52. Such dichotomous functionality underscores the immense therapeutic potential of targeting NLRP3 as a novel strategy for controlling pathological inflammation 53. In recent years, pharmacological advances have led to the identification of several promising small-molecule NLRP3 inhibitors, including MCC950, OLT1177, NT-0796, oridonin, cardamonin, and scoparone, all of which have demonstrated preclinical efficacy 26, 54-57. However, despite these encouraging developments, no NLRP3-targeted therapy has yet progressed to clinical approval. MCC950, the most advanced candidate to date, exhibited potent anti-inflammatory activity in NLRP3-driven disease models but was discontinued in Phase II trials due to hepatotoxicity. Meanwhile, clinical development of OLT1177 and NT-0796 remains at early stages 53. These limitations highlight a critical unmet need for novel NLRP3 inhibitors that can deliver both robust efficacy and acceptable safety profiles.

Emodin, a naturally occurring anthraquinone derivative primarily extracted from traditional medicinal plants such as *Rheum palmatum* (Radix Rhei) and *Polygonum cuspidatum* (Polygonaceae), has been widely recognized in East Asian ethnomedicine. In recent years, it has garnered significant scientific interest due to its potent anti-inflammatory properties 4, 37, 58, 59. Preliminary studies have suggested that emodin may suppress NLRP3 inflammasome activation and reduce IL-1β production 60-62. Building upon these early findings, our current study provides comprehensive mechanistic insights into emodin's broad-spectrum inhibitory effects on NLRP3 inflammasome activation. Specifically, our data demonstrate that emodin robustly inhibits caspase-1 activation and IL-1β maturation induced by both canonical and noncanonical NLRP3 stimuli in BMDMs. Importantly, this inhibitory effect exhibits high specificity for the NLRP3 inflammasome, as emodin did not impair caspase-1 activation or IL-1β secretion mediated by the AIM2 or NLRC4 inflammasomes. Moreover, we found that the concentrations of emodin required to suppress NLRP3 activation did not affect NF-κB-dependent priming signals, such as the induction of pro-IL-1β, NLRP3, or TNF-α production in BMDMs. These observations rule out non-specific suppression of inflammasome priming as a mechanism underlying emodin's anti-inflammatory effects 61-63. Mechanistically, our data reveal that emodin targets a critical upstream step in inflammasome assembly. ASC oligomerization is an essential event in the formation and activation of the NLRP3 inflammasome complex. Our findings demonstrate that emodin markedly reduces ASC speck formation and oligomerization in response to NLRP3 activation, suggesting that emodin exerts its anti-inflammatory effects, at least in part, by disrupting inflammasome assembly at this key molecular checkpoint. Collectively, these results establish emodin as a promising candidate for the development of therapeutics aimed at NLRP3-driven inflammatory diseases. Its dual features of broad-spectrum NLRP3 inhibition and inflammasome pathway specificity, combined with its natural origin, highlight emodin's potential as an attractive lead compound. Further investigation into its pharmacodynamic properties, bioavailability, and safety profile is warranted to facilitate its translation into clinical applications.

Beyond their canonical functions in bioenergetics, mitochondria have recently emerged as pivotal regulators of NLRP3 inflammasome activation. They serve not only as structural scaffolds for inflammasome assembly but also as critical sensors of cellular stress. Mitochondrial damage and the subsequent generation of mtROS represent potent triggers for NLRP3 inflammasome assembly, playing indispensable roles in initiating inflammatory signaling cascades 1, 17, 23, 64. This paradigm underscores the therapeutic promise of targeting mitochondrial quality control mechanisms to modulate NLRP3-driven pathologies 65. Central to this quality control network is mitophagy, a specialized autophagy pathway responsible for the selective elimination of damaged mitochondria and excessive mtROS production, thereby acting as a critical brake on NLRP3 inflammasome activation 1. Our RNA-seq analysis revealed a robust correlation between emodin-mediated NLRP3 suppression and the modulation of mitochondrial homeostasis and mitophagy pathways. Functional validation demonstrated that emodin significantly attenuated mitochondrial damage and mtROS accumulation in BMDMs stimulated with LPS and nigericin, thereby preserving mitochondrial integrity. Furthermore, emodin enhanced mitophagy flux, as evidenced by elevated LC3-II/I ratios and increased co-localization of damaged mitochondria with autophagosomes. Importantly, pharmacological inhibition of mitophagy using 3-MA abolished emodin's protective effects on mitochondrial integrity and reversed its inhibitory action on NLRP3 activation in stimulated BMDMs. Collectively, these findings establish a causal link between emodin-induced mitophagy and NLRP3 inflammasome inactivation, proposing a mechanistic axis wherein emodin disrupts the pathological cycle of mitochondrial damage, mtROS production, and inflammasome activation via enhanced mitophagy.

Mitophagy, a meticulously regulated cellular process governed by intricate molecular crosstalk, serves as a linchpin for maintaining mitochondrial homeostasis under physiological and pathophysiological conditions 47, 66, 67. Among the diverse mitophagy pathways, the FUNDC1-dependent axis emerges as a highly dynamic stress-responsive mechanism, critically contributing to mitochondrial quality surveillance and suppression of NLRP3 inflammasome activation 68. This mitochondrial outer membrane protein functions as a mitophagy receptor through direct interaction with microtubule-associated protein LC3 69. Central to this process is the phosphorylation status of FUNDC1: CK2-mediated phosphorylation at Ser13 disrupts FUNDC1-LC3 binding, thereby suppressing mitophagy flux. Conversely, dephosphorylation of FUNDC1 restores mitophagy, underscoring this post-translational modification (PTM) as a critical molecular switch for mitophagy regulation 31, 36, 70. Notably, recent pharmacological investigations have identified emodin as a selective CK2 inhibitor that competitively occupies the ATP-binding pocket of the kinase 71. Building on these observations, we demonstrated that emodin dose-dependently attenuated CK2 enzymatic activity in BMDMs and formed stable complexes with CK2, while exhibiting negligible off-target interactions with FUNDC1 or LC3. These findings collectively implicate emodin as a potential modulator of FUNDC1-dependent mitophagy through disruption of CK2-mediated phosphorylation events. Furthermore, functional validation revealed that emodin treatment elicited rapid FUNDC1 dephosphorylation, augmented FUNDC1-LC3 co-immunoprecipitation, and enhanced mitochondrial-LC3 colocalization. Silencing FUNDC1 significantly abrogated emodin-mediated preservation of mitochondrial integrity and reinstated NLRP3 inflammasome activation, thereby substantiating the functional primacy of FUNDC1-dependent mitophagy in these processes. Integrating these observations, our study uncovers a novel regulatory paradigm wherein emodin suppresses CK2 activity, promoting FUNDC1 dephosphorylation and mitophagy flux. This cascade restores mitochondrial homeostasis while concomitantly attenuating NLRP3 inflammasome activation, highlighting a previously unrecognized role for CK2-FUNDC1 axis modulation in inflammation resolution. Remarkably, this PTM-driven regulatory mechanism operates with exceptional temporal efficiency, mirroring the rapid activation kinetics of the NLRP3 inflammasome.

Our *in vitro* studies have elucidated a novel mechanism by which emodin suppresses NLRP3 inflammasome activation, specifically through the modulation of FUNDC1-mediated mitophagy. To assess the translational relevance of these findings, we employed a murine sepsis model, induced by LPS-triggered NLRP3 inflammasome activation. Emodin administration demonstrated robust anti-inflammatory efficacy, as evidenced by significantly reduced pro-inflammatory cytokine levels and attenuated histopathological injuries in both pulmonary and hepatic tissues. Importantly, emodin conferred a marked survival advantage in mice challenged with lethal doses of LPS, emphasizing its protective capacity in sepsis pathogenesis. Crucially, emodin exhibited minimal impact on inflammatory cytokine profiles, tissue damage, or survival outcomes in NLRP3-deficient mice. This genetic specificity highlights that emodin's beneficial effects in LPS-induced sepsis are fundamentally reliant on NLRP3 inflammasome activation. To further investigate the mechanistic basis of emodin's action, we utilized macrophage-targeted AAV-mediated knockdown of FUNDC1. This genetic intervention abolished emodin's ability to induce mitophagy, suppress NLRP3 inflammasome activation, and improve survival in septic mice. These findings not only validate emodin's therapeutic potential in NLRP3-driven sepsis but also establish FUNDC1-mediated mitophagy as a crucial effector pathway. The convergence of *in vitro* mechanistic insights and *in vivo* validation strongly supports the development of emodin-based strategies for targeting NLRP3-driven inflammatory diseases.

Despite the promising findings presented in this study, several important limitations should be acknowledged. First, although we demonstrated that emodin suppresses NLRP3 inflammasome activation and promotes FUNDC1-mediated mitophagy, these results were primarily derived from animal models and *ex vivo* analyses using human PBMCs. The clinical relevance of these observations remains to be validated in human disease contexts. Second, the pharmacokinetic properties of emodin, particularly its poor oral bioavailability and metabolic instability, pose significant challenges to its therapeutic development. Moreover, the toxicological mechanisms of emodin are not yet fully understood and require further investigation. Future studies should focus on structural optimization of emodin or development of targeted delivery systems to improve its bioavailability and safety profile. Finally, well-designed clinical studies are essential to determine the feasibility and efficacy of emodin-based interventions in inflammatory diseases. Addressing these challenges will be critical for translating our findings into potential clinical applications.

## Conclusion

In summary, our findings elucidate a novel mechanism through which emodin exerts potent suppression of NLRP3 inflammasome activation. Specifically, emodin enhances mitochondrial quality control by promoting mitophagy via CK2-mediated dephosphorylation of FUNDC1. Functionally, emodin demonstrated robust protective effects in LPS-induced septic shock, whereas these effects were abolished in NLRP3-deficient mice or upon macrophage-specific deletion of FUNDC1. Collectively, these results not only establish the molecular underpinnings of emodin's anti-inflammatory activity, but also highlight its therapeutic potential in treating NLRP3-driven inflammatory and infectious diseases. Further investigation of emodin's pharmacological properties is warranted to explore its translational potential in clinical settings.

## Supplementary Material

Supplementary figures and tables.

## References

[1] Swanson KV, Deng M, Ting JP (2019). The NLRP3 inflammasome: molecular activation and regulation to therapeutics. Nat Rev Immunol.

[2] Zhan X, Li Q, Xu G, Xiao X, Bai Z (2022). The mechanism of NLRP3 inflammasome activation and its pharmacological inhibitors. Front Immunol.

[3] Franchi L, Eigenbrod T, Núñez G (2009). Cutting edge: TNF-alpha mediates sensitization to ATP and silica via the NLRP3 inflammasome in the absence of microbial stimulation. J Immunol.

[4] Bauernfeind FG, Horvath G, Stutz A, Alnemri ES, MacDonald K, Speert D (2009). Cutting edge: NF-kappaB activating pattern recognition and cytokine receptors license NLRP3 inflammasome activation by regulating NLRP3 expression. J Immunol.

[5] Xing Y, Yao X, Li H, Xue G, Guo Q, Yang G (2017). Cutting Edge: TRAF6 Mediates TLR/IL-1R Signaling-Induced Nontranscriptional Priming of the NLRP3 Inflammasome. J Immunol.

[6] Mangan MSJ, Olhava EJ, Roush WR, Seidel HM, Glick GD, Latz E (2018). Targeting the NLRP3 inflammasome in inflammatory diseases. Nat Rev Drug Discov.

[7] Chen G, Shaw MH, Kim YG, Nuñez G (2009). NOD-like receptors: role in innate immunity and inflammatory disease. Annu Rev Pathol.

[8] Fu J, Wu H (2023). Structural Mechanisms of NLRP3 Inflammasome Assembly and Activation. Annu Rev Immunol.

[9] Xia CY, Guo YX, Lian WW, Yan Y, Ma BZ, Cheng YC (2023). The NLRP3 inflammasome in depression: Potential mechanisms and therapies. Pharmacol Res.

[10] Jo EK, Kim JK, Shin DM, Sasakawa C (2016). Molecular mechanisms regulating NLRP3 inflammasome activation. Cell Mol Immunol.

[11] Fernandes-Alnemri T, Wu J, Yu JW, Datta P, Miller B, Jankowski W (2007). The pyroptosome: a supramolecular assembly of ASC dimers mediating inflammatory cell death via caspase-1 activation. Cell Death Differ.

[12] Heneka MT, Kummer MP, Stutz A, Delekate A, Schwartz S, Vieira-Saecker A (2013). NLRP3 is activated in Alzheimer's disease and contributes to pathology in APP/PS1 mice. Nature.

[13] Wen H, Gris D, Lei Y, Jha S, Zhang L, Huang MT (2011). Fatty acid-induced NLRP3-ASC inflammasome activation interferes with insulin signaling. Nat Immunol.

[14] Chen S, Sun B (2013). Negative regulation of NLRP3 inflammasome signaling. Protein Cell.

[15] Li Y, Yu J, Li R, Zhou H, Chang X (2024). New insights into the role of mitochondrial metabolic dysregulation and immune infiltration in septic cardiomyopathy by integrated bioinformatics analysis and experimental validation. Cell Mol Biol Lett.

[16] Baik SH, Ramanujan VK, Becker C, Fett S, Underhill DM, Wolf AJ (2023). Hexokinase dissociation from mitochondria promotes oligomerization of VDAC that facilitates NLRP3 inflammasome assembly and activation. Sci Immunol.

[17] Xian H, Watari K, Sanchez-Lopez E, Offenberger J, Onyuru J, Sampath H (2022). Oxidized DNA fragments exit mitochondria via mPTP- and VDAC-dependent channels to activate NLRP3 inflammasome and interferon signaling. Immunity.

[18] Su L, Zhang J, Gomez H, Kellum JA, Peng Z (2023). Mitochondria ROS and mitophagy in acute kidney injury. Autophagy.

[19] Hou Y, Wang Q, Han B, Chen Y, Qiao X, Wang L (2021). CD36 promotes NLRP3 inflammasome activation via the mtROS pathway in renal tubular epithelial cells of diabetic kidneys. Cell Death Dis.

[20] Biasizzo M, Kopitar-Jerala N (2020). Interplay Between NLRP3 Inflammasome and Autophagy. Front Immunol.

[21] Zhong Z, Umemura A, Sanchez-Lopez E, Liang S, Shalapour S, Wong J (2016). NF-κB Restricts Inflammasome Activation via Elimination of Damaged Mitochondria. Cell.

[22] Jiang T, Liu E, Li Z, Yan C, Zhang X, Guan J (2024). SIRT1-Rab7 axis attenuates NLRP3 and STING activation through late endosomal-dependent mitophagy during sepsis-induced acute lung injury. Int J Surg.

[23] Chang X, Liu R, Li R, Peng Y, Zhu P, Zhou H (2023). Molecular Mechanisms of Mitochondrial Quality Control in Ischemic Cardiomyopathy. Int J Biol Sci.

[24] Pang B, Dong G, Pang T, Sun X, Liu X, Nie Y (2024). Emerging insights into the pathogenesis and therapeutic strategies for vascular endothelial injury-associated diseases: focus on mitochondrial dysfunction. Angiogenesis.

[25] Luo T, Jia X, Feng WD, Wang JY, Xie F, Kong LD (2023). Bergapten inhibits NLRP3 inflammasome activation and pyroptosis via promoting mitophagy. Acta Pharmacol Sin.

[26] Feng WD, Wang Y, Luo T, Jia X, Cheng CQ, Wang HJ (2023). Scoparone suppresses mitophagy-mediated NLRP3 inflammasome activation in inflammatory diseases. Acta Pharmacol Sin.

[27] Kuang Y, Ma K, Zhou C, Ding P, Zhu Y, Chen Q (2016). Structural basis for the phosphorylation of FUNDC1 LIR as a molecular switch of mitophagy. Autophagy.

[28] Onishi M, Yamano K, Sato M, Matsuda N, Okamoto K (2021). Molecular mechanisms and physiological functions of mitophagy. Embo j.

[29] Zhang X, Zhou H, Chang X (2023). Involvement of mitochondrial dynamics and mitophagy in diabetic endothelial dysfunction and cardiac microvascular injury. Arch Toxicol.

[30] Chang X, Li Y, Liu J, Wang Y, Guan X, Wu Q (2023). ß-tubulin contributes to Tongyang Huoxue decoction-induced protection against hypoxia/reoxygenation-induced injury of sinoatrial node cells through SIRT1-mediated regulation of mitochondrial quality surveillance. Phytomedicine.

[31] Chen G, Han Z, Feng D, Chen Y, Chen L, Wu H (2014). A regulatory signaling loop comprising the PGAM5 phosphatase and CK2 controls receptor-mediated mitophagy. Mol Cell.

[32] Liu L, Feng D, Chen G, Chen M, Zheng Q, Song P (2012). Mitochondrial outer-membrane protein FUNDC1 mediates hypoxia-induced mitophagy in mammalian cells. Nat Cell Biol.

[33] Bi Y, Liu S, Qin X, Abudureyimu M, Wang L, Zou R (2024). FUNDC1 interacts with GPx4 to govern hepatic ferroptosis and fibrotic injury through a mitophagy-dependent manner. J Adv Res.

[34] Wang Y, Jasper H, Toan S, Muid D, Chang X, Zhou H (2021). Mitophagy coordinates the mitochondrial unfolded protein response to attenuate inflammation-mediated myocardial injury. Redox Biol.

[35] Cai C, Guo Z, Chang X, Li Z, Wu F, He J (2022). Empagliflozin attenuates cardiac microvascular ischemia/reperfusion through activating the AMPKα1/ULK1/FUNDC1/mitophagy pathway. Redox Biol.

[36] Liu L, Sakakibara K, Chen Q, Okamoto K (2014). Receptor-mediated mitophagy in yeast and mammalian systems. Cell Res.

[37] Dong X, Fu J, Yin X, Cao S, Li X, Lin L (2016). Emodin: A Review of its Pharmacology, Toxicity and Pharmacokinetics. Phytother Res.

[38] Liu Y, Shang L, Zhou J, Pan G, Zhou F, Yang S (2022). Emodin Attenuates LPS-Induced Acute Lung Injury by Inhibiting NLRP3 Inflammasome-Dependent Pyroptosis Signaling Pathway *In vitro* and *In vivo*. Inflammation.

[39] Mitra S, Anjum J, Muni M, Das R, Rauf A, Islam F (2022). Exploring the journey of emodin as a potential neuroprotective agent: Novel therapeutic insights with molecular mechanism of action. Biomed Pharmacother.

[40] Guo R, Li Y, Han M, Liu J, Sun Y (2020). Emodin attenuates acute lung injury in Cecal-ligation and puncture rats. Int Immunopharmacol.

[41] Yang Y, Xu J, Tu J, Sun Y, Zhang C, Qiu Z (2024). Polygonum cuspidatum Sieb. et Zucc. Extracts improve sepsis-associated acute kidney injury by inhibiting NF-κB-mediated inflammation and pyroptosis. J Ethnopharmacol.

[42] Han JW, Shim DW, Shin WY, Heo KH, Kwak SB, Sim EJ (2015). Anti-inflammatory effect of emodin via attenuation of NLRP3 inflammasome activation. Int J Mol Sci.

[43] Sarno S, Moro S, Meggio F, Zagotto G, Dal Ben D, Ghisellini P (2002). Toward the rational design of protein kinase casein kinase-2 inhibitors. Pharmacol Ther.

[44] Battistutta R, Sarno S, De Moliner E, Papinutto E, Zanotti G, Pinna LA (2000). The replacement of ATP by the competitive inhibitor emodin induces conformational modifications in the catalytic site of protein kinase CK2. J Biol Chem.

[45] Broz P, Pelegrín P, Shao F (2020). The gasdermins, a protein family executing cell death and inflammation. Nat Rev Immunol.

[46] Lin Q, Li S, Jiang N, Shao X, Zhang M, Jin H (2019). PINK1-parkin pathway of mitophagy protects against contrast-induced acute kidney injury via decreasing mitochondrial ROS and NLRP3 inflammasome activation. Redox Biol.

[47] Lu Y, Li Z, Zhang S, Zhang T, Liu Y, Zhang L (2023). Cellular mitophagy: Mechanism, roles in diseases and small molecule pharmacological regulation. Theranostics.

[48] Baechler BL, Bloemberg D, Quadrilatero J (2019). Mitophagy regulates mitochondrial network signaling, oxidative stress, and apoptosis during myoblast differentiation. Autophagy.

[49] Mizushima N, Yoshimori T (2007). How to interpret LC3 immunoblotting. Autophagy.

[50] Wang S, Long H, Hou L, Feng B, Ma Z, Wu Y (2023). The mitophagy pathway and its implications in human diseases. Signal Transduct Target Ther.

[51] Hsu CG, Li W, Sowden M, Chávez CL, Berk BC (2023). Pnpt1 mediates NLRP3 inflammasome activation by MAVS and metabolic reprogramming in macrophages. Cell Mol Immunol.

[52] Pfalzgraff A, Weindl G (2019). Intracellular Lipopolysaccharide Sensing as a Potential Therapeutic Target for Sepsis. Trends Pharmacol Sci.

[53] Coll RC, Schroder K, Pelegrín P (2022). NLRP3 and pyroptosis blockers for treating inflammatory diseases. Trends Pharmacol Sci.

[54] Coll RC, Robertson AA, Chae JJ, Higgins SC, Muñoz-Planillo R, Inserra MC (2015). A small-molecule inhibitor of the NLRP3 inflammasome for the treatment of inflammatory diseases. Nat Med.

[55] He H, Jiang H, Chen Y, Ye J, Wang A, Wang C (2018). Oridonin is a covalent NLRP3 inhibitor with strong anti-inflammasome activity. Nat Commun.

[56] Wang Z, Xu G, Gao Y, Zhan X, Qin N, Fu S (2019). Cardamonin from a medicinal herb protects against LPS-induced septic shock by suppressing NLRP3 inflammasome. Acta Pharm Sin B.

[57] Yang Y, Wang H, Kouadir M, Song H, Shi F (2019). Recent advances in the mechanisms of NLRP3 inflammasome activation and its inhibitors. Cell Death Dis.

[58] Song B, Zeng Y, Cao Y, Zhang J, Xu C, Pan Y (2023). Emerging role of METTL3 in inflammatory diseases: mechanisms and therapeutic applications. Front Immunol.

[59] Su J, Xiao J, Deng X, Lin X, Xie L, Ye H (2024). Combining Aloin with TIENAM ameliorates cecal ligation and puncture-induced sepsis in mice by attenuating inflammation and modulating abdominal cavity microbiota. Int Immunopharmacol.

[60] De Gaetano A, Solodka K, Zanini G, Selleri V, Mattioli AV, Nasi M (2021). Molecular Mechanisms of mtDNA-Mediated Inflammation. Cells.

[61] Kumar A, Dhawan S, Aggarwal BB (1998). Emodin (3-methyl-1,6,8-trihydroxyanthraquinone) inhibits TNF-induced NF-kappaB activation, IkappaB degradation, and expression of cell surface adhesion proteins in human vascular endothelial cells. Oncogene.

[62] Xian M, Cai J, Zheng K, Liu Q, Liu Y, Lin H (2021). Aloe-emodin prevents nerve injury and neuroinflammation caused by ischemic stroke via the PI3K/AKT/mTOR and NF-κB pathway. Food Funct.

[63] Chen P, Huang NY, Pang B, Ye ZJ, Luo RX, Liu C (2023). Proteomic and metabolomic approaches elucidate the molecular mechanism of emodin against neuropathic pain through modulating the gamma-aminobutyric acid (GABA)-ergic pathway and PI3K/AKT/NF-κB pathway. Phytother Res.

[64] Chang X, Zhang Q, Huang Y, Liu J, Wang Y, Guan X (2024). Quercetin inhibits necroptosis in cardiomyocytes after ischemia-reperfusion via DNA-PKcs-SIRT5-orchestrated mitochondrial quality control. Phytother Res.

[65] Chang X, Zhou S, Liu J, Wang Y, Guan X, Wu Q (2024). Zishen Tongyang Huoxue decoction (TYHX) alleviates sinoatrial node cell ischemia/reperfusion injury by directing mitochondrial quality control via the VDAC1-β-tubulin signaling axis. J Ethnopharmacol.

[66] Chang X, Zhou S, Liu J, Wang Y, Guan X, Wu Q (2024). Zishenhuoxue decoction-induced myocardial protection against ischemic injury through TMBIM6-VDAC1-mediated regulation of calcium homeostasis and mitochondrial quality surveillance. Phytomedicine.

[67] Zeng Z, Zhou X, Wang Y, Cao H, Guo J, Wang P (2022). Mitophagy-A New Target of Bone Disease. Biomolecules.

[68] Li J, Yang D, Li Z, Zhao M, Wang D, Sun Z (2023). PINK1/Parkin-mediated mitophagy in neurodegenerative diseases. Ageing Res Rev.

[69] Chen M, Chen Z, Wang Y, Tan Z, Zhu C, Li Y (2016). Mitophagy receptor FUNDC1 regulates mitochondrial dynamics and mitophagy. Autophagy.

[70] Kim BH, Koh HC (2023). The role of CK2 in the regulation of mitochondrial autophagy induced by rotenone. Toxicol Lett.

[71] Shrimali D, Shanmugam MK, Kumar AP, Zhang J, Tan BK, Ahn KS (2013). Targeted abrogation of diverse signal transduction cascades by emodin for the treatment of inflammatory disorders and cancer. Cancer Lett.

