# Supplementary material for: Emodin Inhibits NLRP3 Inflammasome Activation and Protects Against Sepsis via Promoting FUNDC1-Mediated Mitophagy
Source: Int J Biol Sci. 2025 May 27;21(8):3631–48. doi: 10.7150/ijbs.110904 (PMC12160860; doi:10.7150/ijbs.110904)
Supplement: Supplementary file 1 — Supplementary figures and tables. [file ijbsv21p3631s1.pdf]

Supporting Information

1. Materials and Methods

1.1. Enzyme-Linked Immunosorbent Assay (ELISA)

According to the manufacturer's instructions provided by Dakewei (Beijing, China), we utilized ELISA kits for the quantitative measurement of mouse or human IL-1β levels, as well as TNF-α levels in the collected conditioned media or serum samples.

1.2. Lactate Dehydrogenase (LDH) Assay

The release of LDH from BMDMs or PBMCs challenged with inflammasome stimulants in the presence of emodin was quantified using the LDH Cytotoxicity Assay Kit (C0016, Beyotime), adhering to the manufacturer's instructions.

1.3. Western blotting and co-immunoprecipitation

We employed a previously established method to extract proteins from cell culture supernatants. Briefly, cell culture supernatants (Sup) were collected and subjected to centrifugation to remove cellular debris, including dead cells. Protein content in the Sup was concentrated using Amicon® Ultra centrifugal filters (Millipore, UFC801096; 10 kDa cutoff), in accordance with the manufacturer's instructions. Cells or lung tissues were lysed using RIPA buffer, which was supplemented with protease and phosphatase inhibitors. Protein concentrations were determined using the Micro BCA Protein Assay Kit (Pulilai Gene Technology, Beijing, China). An equal amount of protein was separated by SDS-PAGE using polyacrylamide TGX gels (Bio-Rad) and then transferred to polyvinylidene difluoride (PVDF) membranes. After blocking, the PVDF membranes were incubated with specific primary antibodies in TBST at 4°C overnight, washed, and then incubated with the corresponding horseradish peroxidase (HRP)-conjugated secondary antibodies for 1 h at room temperature. The immunoreactive bands were visualized using enhanced chemiluminescence (ECL) and analyzed with the Gel View 6000Plus imaging system (Boluteng Biotechnology, Guangzhou, China). For co-immunoprecipitation, cell lysates treated as described in the "inflammasome stimulation" section were incubated with antibodies overnight at 4°C, and then immunoprecipitated with protein A+G agarose beads (P2012, Beyotime) at 4°C for 6 h. The beads were sedimented, washed five times with washing buffer, resuspended in elution buffer, and then the proteins were eluted by boiling in sample buffer prior to SDS-PAGE electrophoresis.

1.4. ASC oligomerization

BMDMs were seeded in a 25T flask at a density of 1×10^6/ml for cell culture. The next day, the medium was replaced, and cells were primed with 50 ng/ml LPS for 4 h. After priming, the cells were treated with emodin for 1 h, followed by stimulation with nigericin for 30 min. The supernatants were removed, and the cells were rinsed in ice-cold PBS, before being lysed by NP-40 for 30 min. The cell lysates were then

centrifuged at 330g for 10 min at 4°C. The resulting precipitates were washed twice with 1ml of ice-cold PBS and resuspended in 500µl of PBS. Disuccinimidyl suberate (DSS) at a concentration of 2mM was added to the resuspended precipitates and incubated at room temperature with rotation for 30 min. Subsequently, the samples were centrifuged again at 330g for 10 min at 4°C. The cross-linked precipitates were resuspended in 60µl of sample buffer, boiled, and subsequently analyzed by immunoblotting.

1.5. Flow cytometric analysis

To quantify the mitochondrial mass, the cells were stained with 150 nM MitoTracker Deep Red and 100 nM MitoTracker Green for 30 min. Following staining, the cells were washed and resuspended in 500µL of PBS containing 2% FBS for fluorescence-activated cell sorting (FACS) analysis.

1.6. Confocal microscopy

To observe the formation of ASC specks, BMDMs were fixed with 4% paraformaldehyde for 20 min and then permeabilized with 1% Triton X-100 for an additional 20 min. Nonspecific binding sites were blocked by incubating the cells with 5% goat serum. The cells were then incubated with an anti-ASC antibody (1:100 dilution) at 4°C, stained with Alexa Fluor 488 for 1 h, and counterstained with DAPI for 10 min. For the assessment of mitochondrial reactive oxygen species (mtROS) levels, cells were stained with 5 µM MitoSOX for 20 min and counterstained with DAPI for 10 min. The fluorescence intensity was measured and documented using a fluorescence microscope (Olympus, Tokyo, Japan). To detect the colocalization of LC3 and mitochondria, BMDMs were incubated with an anti-LC3B antibody (1:150 dilution), stained with ABflo 555, and counterstained with MitoTracker Green (100 nM). For the investigation of the colocalization of LC3 and FUNDC1, THP-1 cells were transfected with a lentiviral vector expressing Flag-FUNDC1 using Lipofectamine 3000. Three days post-transfection, the cells were ready for subsequent experimentation. THP-1 cells were incubated with an anti-LC3B antibody and anti-FUNDC1 (dilution 1:50), stained with ABflo cy3 and counterstained with ABflo 488. Confocal images were captured using a Carl Zeiss confocal microscope (LSM 980, Berlin, Germany) and processed with the manufacturer's software.

1.7. CK2α ATPase activity assay

Multiple parallel reaction systems were established containing either target compounds or solvent controls to evaluate CK2α ATPase activity. Following 1 h incubation at 37°C, ATP levels in each system were quantified using the CellTiter-Glo assay according to manufacturer specifications. Luminescence intensity measurements were processed through a standard curve fitting algorithm.

1.8. Emodin pull-down assays

Emodin was immobilized onto cyanogen bromide (CNBr)-activated Sepharose 4B matrix via covalent conjugation. THP-1 monocytes were subjected to lysis in RIPA

buffer containing a protease inhibitor cocktail. The lysates were clarified through high-speed centrifugation (12,000 × g, 20 min, 4°C) to obtain particulate-free supernatants. These extracts were incubated with emodin-functionalized affinity beads under constant agitation at 4°C for 16 hours. Following incubation, the bead-bound complexes were subjected to six sequential washing cycles. The retained proteins were subsequently eluted and resolved by SDS-PAGE followed by immunoblotting.

1.9. FUNDC1 siRNA Transfection

THP-1 cells were seeded 12 h before transfection. FUNDC1 small interfering RNAs (siRNA-FUNDC1) were transfected into cells using siRNA transfection reagent (TF20141402, NEOFECT) according to the manufacturer's instruction. FUNDC1 siRNA was purchased from GeneChem (Shanghai, China). The transfected cells were incubated at 37 °C for 24 h. A non-targeting negative stealth siRNA (obtained from GeneChem, Shanghai, China) was used as a negative control.

1.10. Plasmid construction and transfection

For the expression of Flag-tagged FUNDC1, the GV341(Ubi-MCS-3FLAG-SV40-PURO MYCIN) lentivirus expression vector was ordered from GeneChem (Shanghai, China). For lentiviral infection, THP-1 cells were infected with GV341 lentivirus expression vector (MOI = 30) according to the manufacturer’s instructions. After 72 h, the cells were subjected to the subsequent experiment.

2. Supporting Figure

Fig. S1

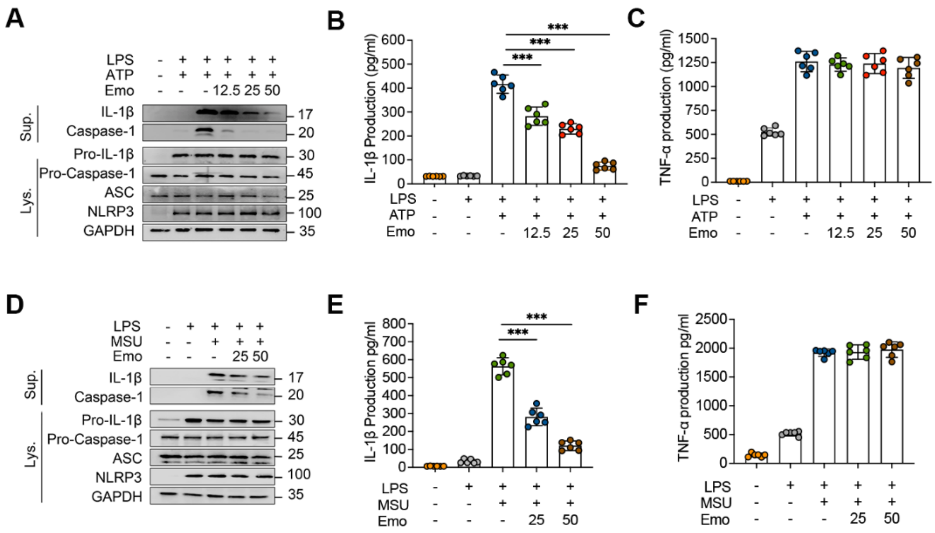

Fig.S1. Emodin inhibits NLRP3 inflammasome activation in LPS-primed BMDMs stimulated with ATP or MSU. (A-C) LPS-primed BMDMs were treated with different concentrations of emodin or vehicle, and then stimulated with ATP. Western blot analysis was performed to detect IL-1β (p17), caspase-1 (p20) in the culture Sup and pro-IL-1β (p30), caspase-1 (p45), NLRP3, and ASC in Lys (A). The secretion of IL-1β (B) and LDH (C) was measured in Sup of BMDMs. (D-F) LPS-primed BMDMs were treated with varying dose of emodin or vehicle, and then stimulated with MSU. Western blot analysis was conducted to assess IL-1β (p17), caspase-1 (p20) in the culture Sup and pro-IL-1β (p30), caspase-1 (p45), NLRP3, and ASC in Lys (D). The secretion of IL-1β (E) and LDH (F) was quantified in Sup of BMDMs. Data are expressed as mean ± SEM from three independent experiments with biological duplicates in (B, C, E, F) or are representative of three independent experiments (A, D). ***P<0.001 indicating significance compared to the control.

Fig. S2

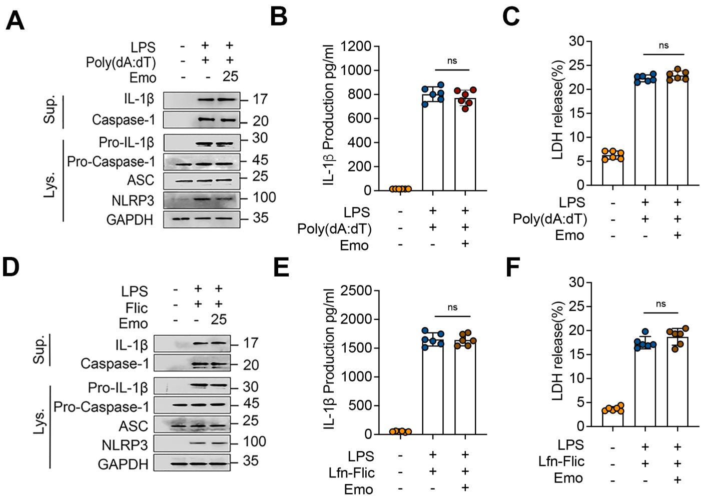

Fig.S2. Emodin exerts no inhibitory effect on AIM2 and NLRC4 inflammasome activation. (A-C) BMDMs primed with LPS were treated with emodin (25 μM) or vehicle, followed by stimulation with poly(dA:dT). Western blot analysis was performed to detect IL-1β (p17), caspase-1 (p20) in culture Sup, and pro-IL-1β (p30), caspase-1 (p45), NLRP3, ASC in Lys (A). The secretion of IL-1β (B) and LDH (C) in Sup from BMDMs was assessed. (D-F) BMDMs primed with LPS were treated with emodin (25 μM) or vehicle, followed by stimulation with Lfn-FliC. Western blot analysis was conducted to detect IL-1β (p17), caspase-1 (p20) in Sup, and pro-IL-1β (p30), caspase-1 (p45), NLRP3, ASC in Lys (D). The secretion of IL-1β (E) and LDH (F) in the Sup from BMDMs was assessed. Data are expressed as mean ± SEM (n = 6) with biological duplicates in (B, C, E, F) or are representative of three independent experiments (A, D). *ns*, not significant.

Fig. S3

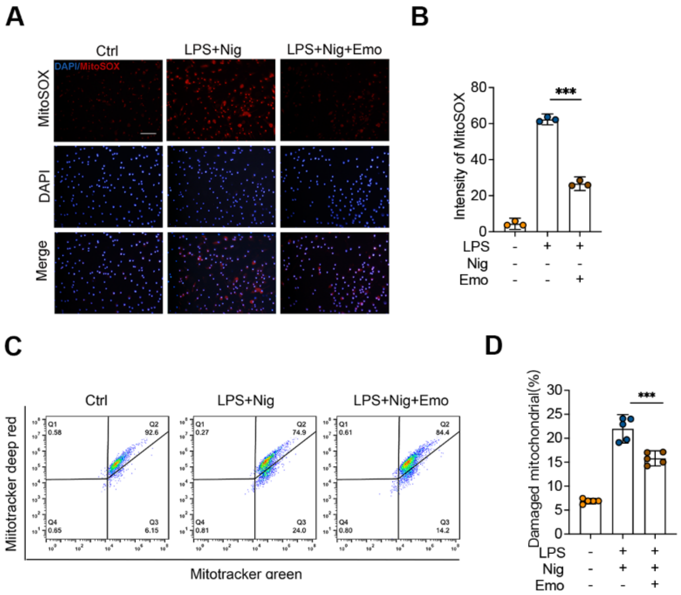

Fig.S3. Emodin enhances mitochondrial homeostasis upon NLRP3 inflammasome activation. LPS-primed BMDMs were treated with emodin or vehicle, and then stimulated with nigericin. (A-B) After treatment, BMDMs were labeled with MitoSOX and analyzed using a fluorescence microscope. Alternatively, BMDMs were stained with Mitotracker Green and Mitotracker Deep Red and analyzed via flow cytometry (C-D). Data are expressed as mean ± SEM from three independent experiments with biological duplicates in (B, D). ****P*<0.001 vs. control.

Fig. S4

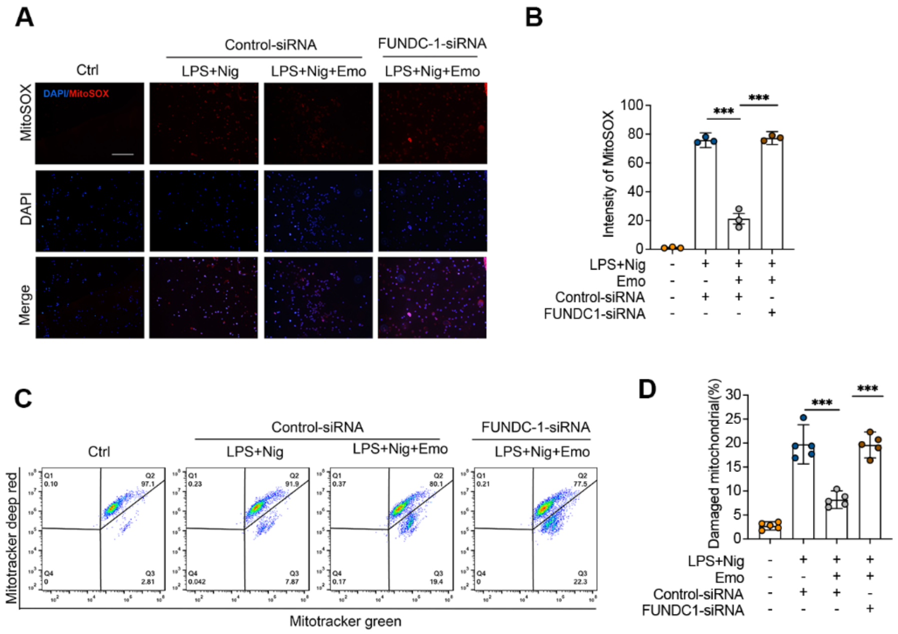

Fig. S4. Emodin promotes FUNDC1-mediated mitophagy. THP-1 cells transfected with siRNA FUNDC1 or control, then pre-conditioned with LPS, were exposed to emodin or a vehicle, followed by nigericin stimulation. Subsequently, the cells were stained with MitoSOX and visualized using fluorescence microscopy (A-B). Alternatively, they were labeled with Mitotracker Green and Mitotracker Deep Red and analyzed through flow cytometry (C-D). Results are reported as the mean ± SEM from three independent experiments, each including biological duplicates for (B, D). ****P*<0.001 denoting significant differences compared to the control.

Fig. S5

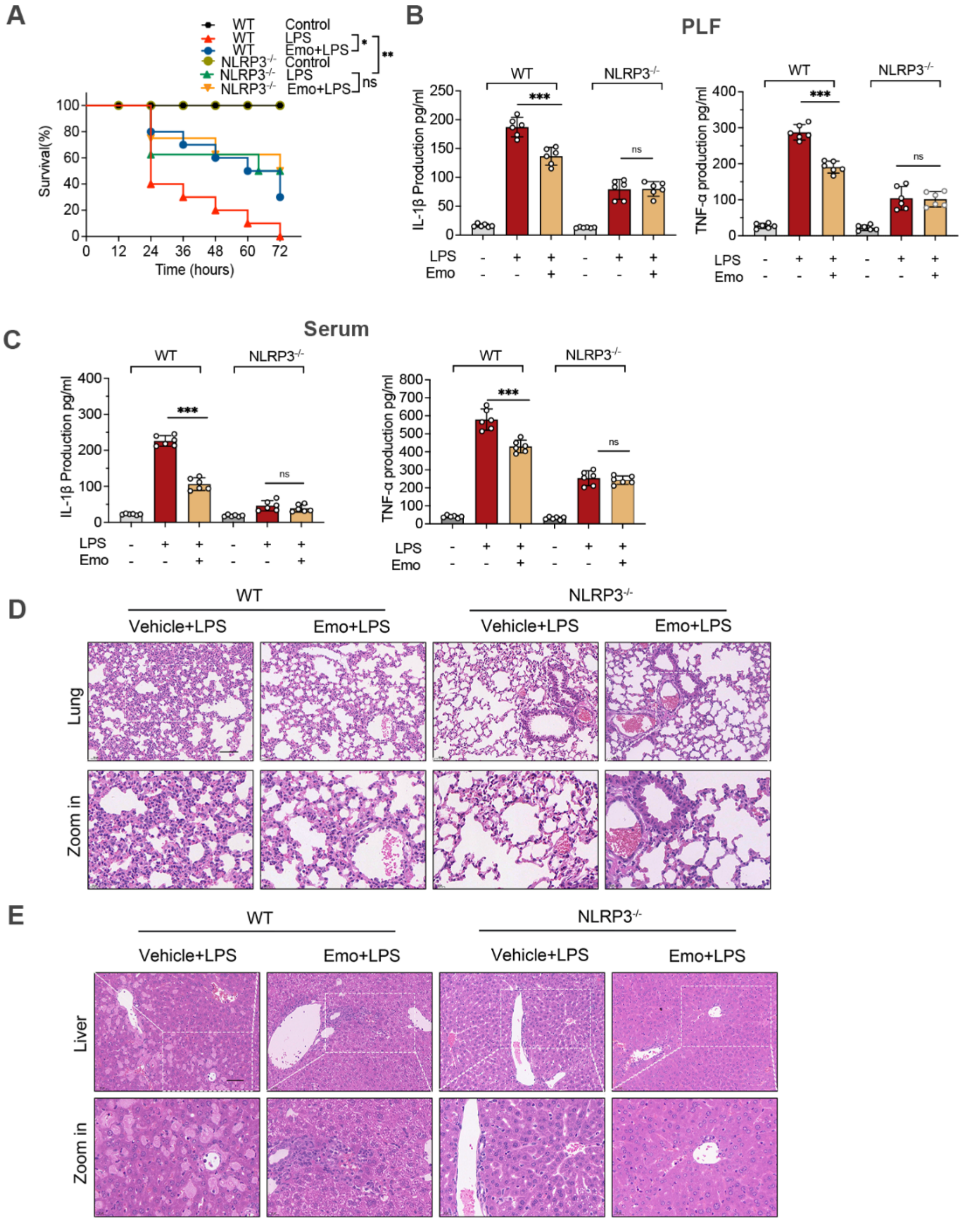

Fig.S5. Emodin protects against LPS-induced sepsis via an NLRP3-dependent mechanism. WT or NLRP3$^{-/-}$ mice were intraperitoneally injected with LPS (10 mg/kg) in the presence or absence of emodin (20 mg/kg). (A) Survival rates were monitored over a 72h period. Statistical significance was determined using the log-rank test. (B-C) Concentrations of IL-1β and TNF-α in serum (B) and PLF (C) were measured by ELISA 8 h after LPS injection. (D-E) Representative microscopic images of H&E-stained lung (D) and liver (E) tissues obtained 24 h post LPS injection. Scale bar, 100 μm. Data are presented as the mean ± SEM. These findings are representative of at least two independent experiments, each including 5-7 animals per group. *P<0.001 vs. control. **P<0.001 vs. control. ***P<0.001 vs. control. *ns*, not significant.
